# The Market Triumph of Ecotourism: An Economic Investigation of the Private and Social Benefits of Competing Land Uses in the Peruvian Amazon

**DOI:** 10.1371/journal.pone.0013015

**Published:** 2010-09-29

**Authors:** Christopher A. Kirkby, Renzo Giudice-Granados, Brett Day, Kerry Turner, Luz Marina Velarde-Andrade, Agusto Dueñas-Dueñas, Juan Carlos Lara-Rivas, Douglas W. Yu

**Affiliations:** 1 State Key Laboratory of Genetic Resources and Evolution, Ecology, Conservation, and Environment Center (ECEC), Kunming Institute of Zoology, Chinese Academy of Science, Kunming, Yunnan, China; 2 School of Biological Sciences, Center for Ecology, Evolution and Conservation (CEEC), University of East Anglia, Norwich, United Kingdom; 3 School of Environmental Sciences, Center for Social and Economic Research on the Global Environment (CSERGE), University of East Anglia, Norwich, United Kingdom; 4 Conservación Ambiental y Desarrollo en el Perú (CAMDE-PERU), Puerto Maldonado, Madre de Dios, Peru; 5 Cooperazione e Sviluppo (CESVI), Puerto Maldonado, Madre de Dios, Peru; 6 Universidad Nacional San Antonio Abad del Cusco (UNSAAC), Puerto Maldonado, Madre de Dios, Peru; University of Western Ontario, Canada

## Abstract

Annual revenue flow to developing countries for ecotourism (or nature-based tourism) could be as large as US$ 210×10^12^, providing an enormous financial incentive against habitat loss and exploitation. However, is ecotourism the most privately and/or socially valuable use of rainforest land? The question is rarely answered because the relevant data, estimates of profits and fixed costs, are rarely available. We present a social cost-benefit analysis of land use in an ecotourism cluster in the Tambopata region of Amazonian Peru. The net present value of ecotourism-controlled land is given by the producer surplus (profits plus fixed costs of ecotourism lodges): US$ 1,158 ha^−1^, which is higher than all currently practiced alternatives, including unsustainable logging, ranching, and agriculture. To our knowledge, this is the first sector-wide study of profitability and producer surplus in a developing-country ecotourism sector and the first to compare against equivalent measures for a spectrum of alternative uses. We also find that ecotourism-controlled land sequesters between 5.3 to 8.7 million tons of above-ground carbon, which is equivalent to between 3000–5000 years of carbon emissions from the domestic component of air and surface travel between the gateway city of Cusco and the lodges, at 2005 emission rates. Ecotourism in Tambopata has successfully monetized the hedonic value of wild nature in Amazonian Peru, and justifies the maintenance of intact rainforest over all alternative uses on narrow economic grounds alone.

## Introduction

Many developing countries still have significant areas of wild nature to showcase for the pleasure of tourists and to attract investment in tourism. In fact, one of the reasons commonly given for justifying the establishment of protected areas (PA) is to profit from *ecotourism*, defined here as ‘travel to natural areas to admire, study or enjoy wild nature in a way that contributes to its conservation and to the wellbeing of local people’ [1]. More broadly, ever since the term “ecotourism” was coined in the 1980s [2], heavy expectations have been placed on its potential to promote conservation and sustainable development [3].

Ecotourists seek out lodgings associated with PAs [4], and indeed the number of visitors to PAs in developing countries is steadily increasing [5]. Thus, ecotourism is expected to bring about (i) economic and job-creation benefits, such as the building, maintenance and operation of hotels, the supply of goods and services to these, and the generation of government tax revenues [6]–[9]; (ii) new educational and training opportunities for management and labor, including interaction with foreigners and others from outside immediate social groups [6], [8], [10]; and (iii) incentives for the conservation of wild nature via the collection of user fees to finance PA management [9], [11]–[13], via economic substitutes for exploitation of natural products, such as hunting [14], [15], and via the establishment of privately-managed reserves on the periphery of PAs [1], [16], [17]. These benefits have led to the funding of innumerable projects and incentive schemes financed by governments, non-governmental organizations (NGOs) and the international community [18]–[20].

However, initial optimism has given way to criticism as (i) the term ecotourism has been appropriated by traditional, even mass tourism, operators, (ii) ecotourism appears not to be economically self-sustaining or providing the expected benefits [19], [21], and (iii) negative impacts of visitors on wildlife and local communities have been documented [8], [22]–[27].

A tour operator is only expected to engage in costly conservation actions under all of the following six conditions: a) if the business is profitable; b) if profits increase with tourist volume; c) if tourists demand high-quality nature (such as an abundance of wildlife or a large expanse of intact forest); d) if the conservation action is expected to be effective and not too costly; e) if additional investments in standard tourist amenities (e.g., hot showers) are of limited value for attracting more tourists; and f) if tourist activities themselves do not cause much environmental damage [1], [17]. Given these conditions, ecotourism ventures often fail on their own terms [19].

However, at least some ecotourism ventures are known to be succeeding [1], [10], [28]–[31], and the case study literature might suffer from selection bias and focus on community-based ventures [19], which are prone to failure. Moreover, top-down estimates suggest that the flow of revenues from developed to developing countries for the purpose of ecotourism could be as great as US$210 billion (×10^12^) per year [1], [32]. In sum, there is reason to believe that ecotourism could indeed and might already be acting as a major promoter of conservation and development [1], with concomitant effects on economies and livelihoods. However, with ecotourism (as with any activity) opportunity costs are exerted on society, in that alternative activities, such as agriculture and livestock ranching, are prevented [33], [34].

This can in turn foment land-use conflict, especially in transitioning frontier areas, where wild nature is most prevalent and seen as an impediment to many forms of development but where governance tends to be weakest [35]. Weak governance structures have the potential to undermine the conservation and development actions of ecotourism activities.

A common response to such conflict is to implement a landscape-level planning strategy, referred to sometimes as *ecological and economic zoning* (EEZ) [36]–[39]. EEZs use knowledge of the land (e.g., soil fertility, slope, available natural resources), of existing economic uses, and of population centers, market demand, transport infrastructure, PAs, and so forth to produce landscape-scale maps with recommended land-use patterns. The objective is productive use of an economically optimal landscape., But EEZs typically do not have access to reliable information on the values of competing land uses. Although market exchange can sometimes reallocate initial land uses so as to achieve efficiency, this does not work for activities that are strongly influenced by land uses that came before. So, ecotourism is unlikely to succeed on former ranchland, and ecotourism must be included in EEZ plans from the outset.

Moreover, ecotourism activities are at particular risk in low governance areas, because the wild nature and intact habitats that they market to their clients are seen by many, particularly locals, as unexploited and privately valueless, and thus ripe for conversion. In areas where ecotourism is (or could be) undertaken, decision makers would benefit from a thorough understanding of the social and private costs and benefits attributable to ecotourism, including opportunity costs from foregone activities.

The geographic focus of this paper is on an area known as Tambopata, Department of Madre de Dios, southeastern Peru, located within the southwest Amazon eco-region and the Tropical Andes biodiversity hotspot. Tambopata is dominated by two PAs, the Tambopata National Reserve (TNR, 274,690 ha, established in 2000) and the Bahuaja-Sonene National Park (BSNP, 1,091,416 ha, established in 1998). In addition to public protected areas and ecotourism, the Tambopata area supports six other major land-uses: (i) swidden agriculture; (ii) cattle ranching; (iii) Brazil nut extraction; (iv) selective timber extraction; (v) alluvial gold mining; and (vi) private reserves [1]. Swidden agriculture and cattle ranching are undertaken on privately controlled, largely titled lands, most of which are close to the regional capital Puerto Maldonado. Extraction of Brazil nuts, gold, and timber is undertaken on state-owned land granted as renewable 40 year concessions to private users, including local families, small- and medium-sized businesses, and conservation-oriented non-governmental organizations for periods of up to 40 years renewable. For some years, logging and gold mining have been the two largest generators of revenue in Madre de Dios, estimated at US$200 million and US$80 million yr^−1^, respectively, and together employ 20,000 people [40]. The agriculture and cattle ranching sectors in Tambopata are small and geared towards both subsistence and supplying the 50,000 people who live in Puerto Maldonado.

Ecotourism has been booming since the mid 1990s, and in 2008, the area boasted 37 ecotourist establishments in and around the TNR, including 100-bed jungle lodges, 30-bed research and education centers and 8-bed family-run home stay guest houses (hereafter, lodges). A companion study [1] has found the ecotourism sector in Tambopata to be generating substantial profits from millions of dollars in revenues, and lodges engage in costly conservation actions, resulting in over 50,000 ha of rainforest coming under or destined for private management.

However, the Tambopata area is threatened by massive deforestation and chaotic development as a consequence of the paving of the Interoceanica Sur (IOS) highway (a westerly extension of the Trans-Amazon Highway, begun in 2005 and due for completion in 2011, Fig. 1). The IOS runs through Tambopata and will increase access to the forest and its resources. Weak governance (policy decision making, interpretation, and implementation) and high prices for commodities such as gold and tropical hardwoods will exacerbate the effect of the IOS [41].

**Figure 1:**
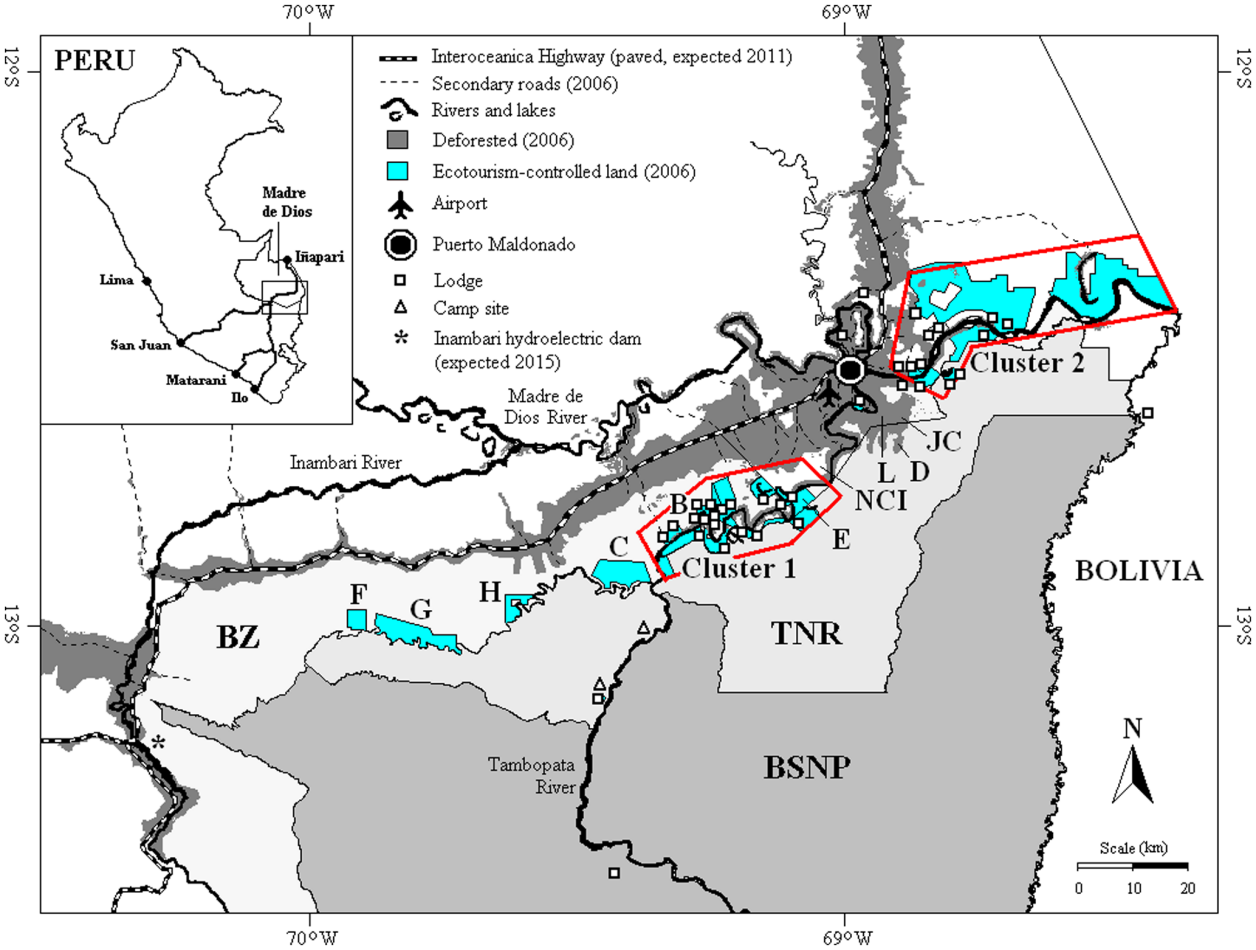
Map of study location. The Tambopata area showing the location of the Tambopata National Reserve (TNR), Bahuaja Sonene National Park (BSNP), their Buffer Zones (BZ), the Interoceanica Highway, secondary roads, ecotourism lodges (white squares), official ecotourism campsites (white triangles), and deforestation up to 2006. ‘D’ denotes 2006 deforestation within the TNR associated with the communities of Jorge Chavez (JC) and Loero (L). Other communities mentioned in the text: Baltimore (B) and Native Community of Infierno (NCI). Most lodges belong to one of two clusters (1 and 2), on the Tambopata River and Madre de Dios River, respectively. Lodge-controlled lands (titled land and ecotourism, conservation and Brazil nut concessions combined, blue) showing the strategic location of two continuous blocks of ecotourism land (enclosed within red lines). These blocks lie between the deforestation fronts associated with both the Interoceanica Highway and the provincial capital of Puerto Maldonado and the limits of the Tambopata National Reserve (TNR) and Bahuaja Sonene National Park (BSNP). The current 20-km wide Jorge-Chavez-Loero gap between the two lodge clusters is centered on L. “C” is a proposed ecotourism concession and “F–H” are ecotourism concessions granted to mestizo communities that have historically been dedicated to mining alluvial gold deposits. “E” is a triangular portion of forested land, located within the Native Community of Infierno, which though not controlled by a lodge has been set aside for their ecotourism joint venture with the Posada Amazonas lodge.

Our premise is that, in low-governance areas such as this, social costs and benefits of activities are rarely internalized by markets or by the state, and land use is determined by private costs and benefits. Under pressure from ecotourism operators, the local judiciary has helped to reverse incursions into ecotourism concessions [1], but this requires that ecotourism be more profitable than alternative uses, or operators would not be incentivized to protect their businesses [1], which returns us to the general expectation that conservation is more likely if the private market value of forest land used for ecotourism sufficiently outweighs the private values realizable from other uses of that land, allowing the market itself to motivate forest preservation.

We therefore endeavor to answer the following questions:

1. Can ecotourism in Tambopata be justified on purely private financial grounds as the most profitable use of land? That is, do the private, financial benefits of ecotourism-controlled forest exceed the opportunity cost of foregone development of the forest?
2. If ecotourism can be justified on private grounds, would the justification still hold up (or even be strengthened) if social costs and benefits were considered?

We achieve this with reference to a set of more specific questions:

1. What are the net-present-values (NPVs) of profits (a measure of private benefits) and producer surplus (a measure of social benefits) from ecotourism activities on forest land controlled by that industry?
2. What are the NPVs of profits and producer surplus on land controlled by alternative land uses, such as Brazil-nut extraction, timber extraction, agriculture, and cattle ranching?
3. How much carbon is sequestered on ecotourism-controlled land (a social benefit), and how much is emitted by tourism (a social cost)?

By answering these questions, we offer decision-makers in Peru the means to assess, ex-post, the economic impact of the original legislation that led to the establishment of PAs in Tambopata and the legislation that encouraged ecotourism development here. Relevant laws include low taxation rates for ecotourism businesses and their recent ability to lease areas of forest for their exclusive use for renewable 40-yr periods. We also offer decision-makers an assessment of the marginal value of intact forest dedicated to ecotourism, as opposed to another activity. Finally, we offer the wider community an insight into the economic value of intact rainforest. This latter topic has been intensively researched and discussed for many years [42]–[51] but has rarely been rigorously estimated with financial data.

Our focus is on producer profits and producer surplus because most ecotourists in Peru are foreign, and their consumer surplus (the hedonic value from having been a tourist) does not therefore accrue to Peru. Only the producer surplus value, particularly the profits component, of ecotourism has the short-term potential to influence land use, as we have shown in a companion study [1]. Despite this, there is a paucity of studies dealing with the private values of tropical forests from the ecotourism and recreational perspective, let alone the distribution of this value amongst local beneficiaries.

We calculate the private NPV per hectare of intact tropical forest land controlled and used by a representative sample of 12 lodges based on their observed private net benefits (i.e. profits) in 2005, the year for which the most complete data set was available. We contrast these land values with the likely foregone development or opportunity costs from alternative uses, including agriculture, cattle ranching, timber extraction, or Brazil nut harvesting. Two timber extraction intensities (sustainable and unsustainable), nine forms of agricultural specialization, and two cattle stocking rates (sustainable and unsustainable) are considered.

We also consider five possible forest development pathways: (i) unsustainable timber extraction followed by permanent agriculture; (ii) unsustainable timber extraction followed by agriculture and subsequently sustainable cattle ranching; (iii) Brazil nut extraction in combination with unsustainable extraction of only high-value timber species (‘high-grading’); (iv) timber ‘high-grading’ followed by ecotourism; and (v) combining ecotourism with Brazil nut extraction. For agriculture, we assume that the distribution of uses in the scenarios is the same as that currently observed. These scenarios are chosen to reflect three observed pathways that are common alternatives to ecotourism (i–iii) and two observed pathways that are compatible with ecotourism (iv–v).

We go on to calculate the carbon emissions from domestic airplane and boat travel by tourists and compare it to the amount of aboveground carbon sequestered by lodges on their privately managed concessions. For lack of data, we do not consider other social benefits of forest cover, such as biodiversity conservation and flood regulation, although these are important justifications for the establishment of protected areas in Peru. We finish with recommendations aimed at policy makers and civil society groups when it comes to land use planning and how they should interpret the results expressed here.

## Results

### Rural household land use

**Figure 2:**
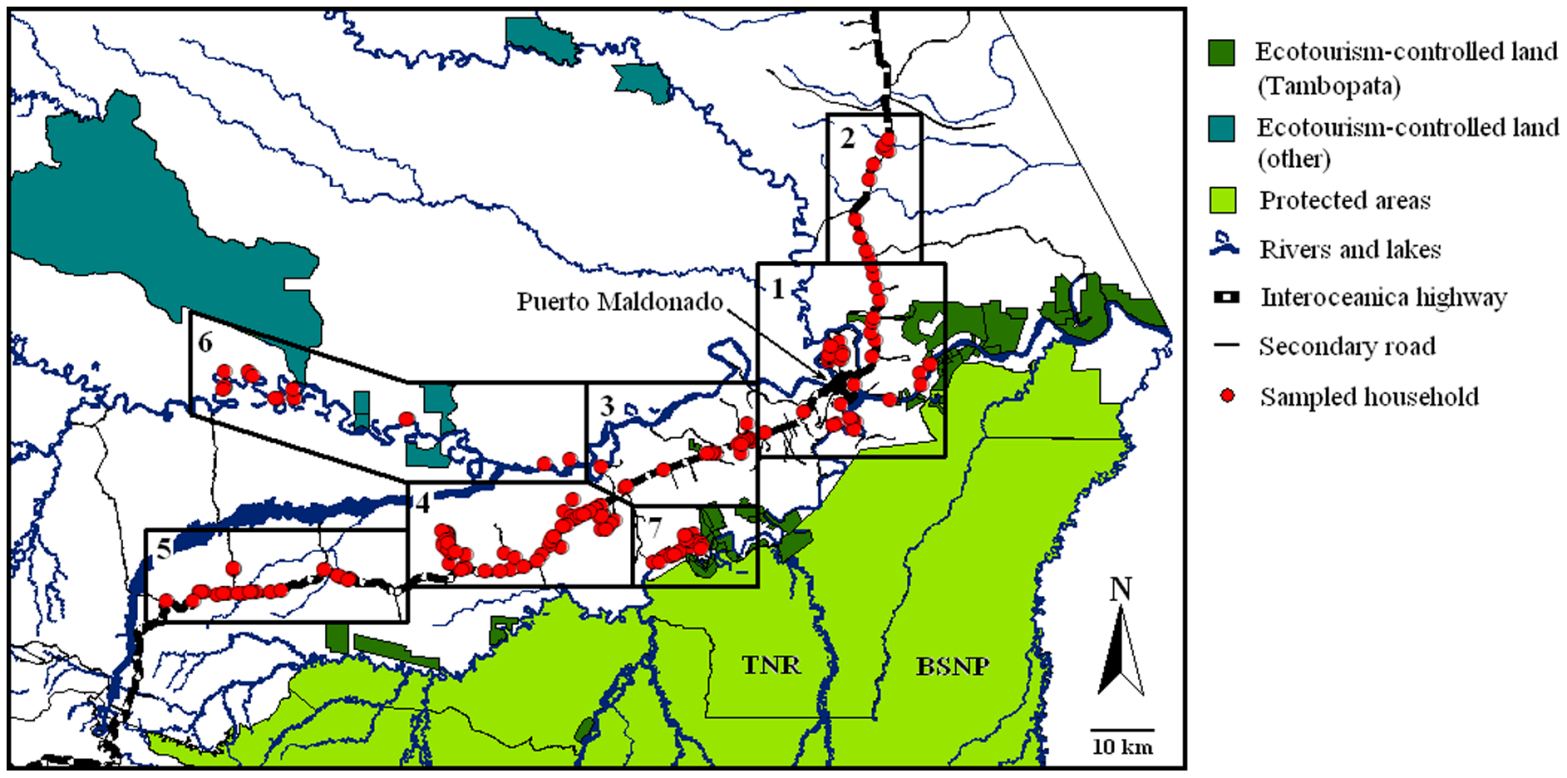
Household interview locations. Map of Tambopata showing the location of the 200 households surveyed in relation to the seven sample areas, the protected areas of Tambopata (TNR, Tambopata National Reserve; BSNP, Bahuaja-Sonene National Park) and associated ecotourism-controlled lands associated with Tambopata (i.e. those within 25 km of the TNR) and those associated with other areas.

Of the 209 rural households questioned regarding the 12-month period corresponding to the 2006–2007 growing season, 200 were economically dependent on the numerous products and resources derived from the lands they owned or managed, either for subsistence or for sale to markets, and did not rely heavily on wage labor. Of these 200, 44 (22%) were located within 1 km of a major navigable river, 91 (46%) within 1 km of the Interoceanica Highway, and 65 (33%) within 1 km of a secondary road or logging track leading to the highway or directly to Puerto Maldonado (Fig. 2). Sampled households controlled land parcels with a mean area of 51.3±4.2 ha (Supporting Information Table S1), of which on average 52.8% was standing forest, 7.8% was cleared for agriculture, 19.9% was cleared for cattle pasture, and the remaining 19.5% was in fallow (mainly secondary forest, locally known as *purma*).

### The amount of ecotourism-controlled versus ecotourism-used land

In total, the twelve lodges in our dataset control 31,807 ha of rainforest (Supporting Information Table S1), of which Reserva Amazonica lodge is an especially large landholder [1], controlling 20,646 ha, including numerous plots of titled land, a tourism concession, a conservation concession, and a Brazil nut concession. Without Reserva Amazonica, the remaining eleven lodges control a mean of 1,015 ha.

Nonetheless, the lodges in total use between 6,611 to 21,160 ha to stage their hikes, depending on whether one includes a 200m visual buffer or an 800m auditory buffer, respectively (Supporting Information Table S1). Given these statistics, it is not surprising that much of the land used to stage walks is actually part of the neighboring reserve (TNR) and not lodge-controlled. The purpose of controlling land, therefore, has much to do with maintaining a natural, forested environment around the lodges themselves, plus capturing options for future expansion and potential REDD projects [1].

The use of the TNR for tourism potentially complicates how we compare the profitability of ecotourism to alternatives. It could be argued that the lodges receive a kind of subsidy from the neighboring TNR, but the lodges help protect the TNR, and entrance fees paid by the lodges fund the local parks budget many times over [1], with the excess transferred to the National Parks service (Servicio Nacional de Areas Naturales Protegidas por el Estado – SERNANP), a dependency of the Ministry of the Environment. We therefore use the amount of ecotourism-controlled land to normalize ecotourism profits and producer surplus (Supporting Information Table S1), reasoning that, in the first instance, this is the land that is being kept from alternative uses and therefore that this is the land for which we should calculate the full suite of alternative valuations. We revisit this issue in the Discussion. We also concentrate on pooled profits, as we are interested in comparing entire sectors and because mean profits are somewhat misleading because some lodges control very little land and therefore result in very high profits per hectare. Note that all profit and producer-surplus values reported below are from 2005 (ecotourism) and 2006 (alternatives). Net present values (NPV) are of course not specific to a year, although they are expressed in units based on the value of money in a particular year.

### Private benefits: Profits

Concentrating first on profits (Supporting Information Table S1), the twelve lodges together generated similar or greater profit (in 2005) ($39 ha^−1^) relative to all alternatives (in 2006), except for a few pig specialists ($47 ha^−1^) who cover only 50 ha total, and unsustainable timber extraction ($158 ha^−1^). The 25-year net present value (NPV) provides a similar picture; ecotourism ($472 ha^−1^) is superior or similar to all but the same two alternatives. Of course, unsustainable timber extraction from ecotourism concessions ($689 ha^−1^) only generates economic activity over the first five years. As a result, while annual mean profits per ha from unsustainable timber harvesting are roughly 4 times those from ecotourism, the NPV of that activity is only 1.5 times as large.

### Social benefits: Producer surplus

The producer surplus of ecotourism ($96 ha^−1^) is two and a half times the profit value of ecotourism ($39 ha^−1^), reflecting the high fixed cost of maintaining an ecotourism operation, and is again higher than almost all alternatives except unsustainable timber extraction ($227 ha^−1^). However, over a 25-year period, ecotourism has the highest NPV producer surplus of all activities ($1158 ha^−1^), even over unsustainable timber extraction ($991 ha^−1^) from ecotourism concessions.

### Combination land uses

Some combinations of activities, including ecotourism, can be run simultaneously or sequentially (Table 1). Does a combination of feasible alternatives outweigh ecotourism? Again, first concentrating on profits, unsustainable timber extraction for five years followed by 20 years of ecotourism results in the highest NPV profit ($991 ha^−1^), even more than a scenario of unsustainable timber extraction followed by agriculture and ranching ($933 ha^−1^). Logging followed by tourism has occurred in the case of Refugio Amazonas lodge in the Condenado area of Tambopata. It is also possible to contemplate removing the most valuable timber in ecotourism concessions, away from the tourist trails, while ecotourism is running.

**Table 1:** NPV of land based on varying the order of land uses.

	Pooled NPV (Profit value)	Mean NPV (Profit value)	Pooled NPV (PS value)	Mean NPV (PS value)
	(US$ ha^−1^)	(US$ ha^−1^)	(US$ ha^−1^)	(US$ ha^−1^)
Timber (high-grading, yrs 1–5) followed by	689	989	990	1,422
Agriculture (all households, yrs 6–25)	204	243	339	395
Total	893	1,232	1,329	1,817
Timber (high-grading, yrs 1–5) followed by	689	989	990	1,422
Agriculture (all households, yrs 6–10) followed by	80	96	134	156
Cattle ranching (sustainable, yrs 11–25)	163	169	285	298
Total	933	1,253	1,408	1,876
Timber (high-grading, yrs 1–5) in conjunction with	689	989	990	1,422
Brazil nuts (yrs 1–25)	76	74	80	79
Total	765	1,063	1,070	1,500
Timber (high-grading, yrs 1–5) followed by	689	989	990	1,422
Ecotourism-controlled land (yrs 6–25)	302	1,996	742	5,491
Total	991	2,985	1,731	6,913
Ecotourism-controlled land (yrs 1–25) in conjunction with	472	3,117	1,158	8,575
Brazil nuts (yrs 1–25)	76	74	80	79
Total	548	3,191	1,238	8,654

From a producer surplus standpoint, the timber to ecotourism scenario continues to generate the highest NPV returns ($1731 ha^−1^), increasing its margin of superiority over the unsustainable timber to agriculture and ranching scenario ($1408 ha^−1^).

### Net carbon emissions from ecotourism

Ecotourism-controlled land contains between 5.3 to 8.7 million tons of above-ground carbon, equivalent to between 3 to 5 thousand years of carbon emissions from tourism, at 2005 rates. The analysis here is limited to emissions from domestic travel (Cusco to Puerto Maldonado by air, followed by bus and boat travel to the lodges) (Table 2). Variation among lodges is driven mainly by the sizes of the land that they control, which vary by two orders of magnitude.

**Table 2:** Carbon emissions and above-ground carbon stocks. For 12 lodges, historical carbon emissions from visitor flights (CUS-PEM-CUS) and fossil fuel consumption, above ground (AG) carbon stocks on lodge-controlled and trail-buffer lands, and years required for emissions to equal stocks assuming constant 2005 emissions rate. Min = Minimum AG carbon estimate (tC/ha); Max = Maximum AG carbon estimate (tC/ha).

LODGE	2005 carbon emissions (tC)	Total carbon emissions since lodge has been open (tC)	2005 stock of AG carbon on lodge-controlled land (tC) Min	2005 stock of AG carbon on lodge-controlled land (tC) Max	Years to equal 2005 stock at 2005 rate Min	Years to equal 2005 stock at 2005 rate Max
Bello Horizonte	10	32	38,678	63,376	3,736	6,123
Casas Hospedaje Baltimore (pooled)	3	37	7,470	12,240	2,325	3,818
Ecoamazonia Lodge	318	1,522	1,039,492	1,703,264	3,264	5,352
Explorer's Inn	108	2,791	17,264	28,288	135	237
Libertador Tambopata Lodge	225	1,292	166,000	272,000	732	1,203
Picaflor Research Centre	4	12	237,380	388,960	54,459	89,236
Posada Amazonas	284	1,641	166,000	272,000	579	952
Reserva Amazonica	310	1,964	3,427,236	5,615,712	11,064	18,132
Sandoval Lake Lodge	211	1,007	22,576	36,992	102	170
Tambopata Research Center	102	833	53,950	88,400	519	856
Taricaya	59	200	88,976	145,792	1,492	2,447
Wasai Lodge	91	422	14,940	24,480	160	265
Total	1,725	11,753	5,279,962	8,651,504	3,053	5,007
Mean	144	979	439,997	720,959	254	417
N	12	12	12	12	12	12

## Discussion

Ecotourism monetizes the hedonic value of wild nature. When most tourists originate from foreign countries, profit and producer surplus are the relevant measures of this hedonic value, given that it is the local producers and destination countries who must pay the opportunity costs of maintaining natural attractions. However, measures of profits and producer surplus are rare in the literature [9], [21], [31], [52], due to the difficulty of gaining access to private financial data. To our knowledge, this is the first sector-wide study of profitability and producer surplus analysis in a developing-country ecotourism sector [1] and the first to compare against equivalent measures for a spectrum of alternative uses.

Despite the rather large amount of data, the results are straightforward. The ecotourism sector in Tambopata, as a group, generates more profit per hectare than any other activity, with the trivial exception of small-scale pig farming, which cannot be scaled up, and the nontrivial exception of selective logging (Supporting Information Table S1).

If a social measure of value, producer surplus, is used, then ecotourism has the highest net present value of all activities, including logging (Supporting Information Table S1), even without assuming further growth in tourist volume. Combining logging with agriculture and ranching can increase total profits and producer surplus per hectare, but less so than combining logging with ecotourism (Table 1). Finally, carbon emissions from domestic air and land travel amount to a tiny proportion of the above-ground carbon sequestered on ecotourism-controlled lands.

Thus, with reference to our motivating questions (Introduction), ecotourism is one of the two most privately profitable uses of rainforest in Tambopata, the other being unsustainable, selective logging, and is more profitable than agriculture. If we use the broader, social measure of value, producer surplus, ecotourism is the single most valuable use of rainforest in Tambopata, because of the high fixed costs inherent in running a tourism operation, which benefit the local economy via wages and spending [1]. Finally, if we assume that the lodges will be able to protect forest cover from deforestation, which is threatened by the paving of the Interoceanica Highway [1], then the lodges have already sequestered far more carbon than they can reasonably expect to emit over the lifetime of their enterprises.

### Caveats, biases, and omissions

Even with the unprecedented depth and scope of the financial data that were made available to us, there are still multiple sources of bias and omissions that must be identified.

#### Profit estimates

Our estimates of the value of ecotourism are conservative in two ways. Many shareholders pay themselves above-market wages for their management activities, which constitutes a second way of extracting profits from tourism operations. It appears that this flow roughly doubles lodge profits, although we caution that our dataset is incomplete [1]. Furthermore, tourist volume and profits for the sector as a whole are currently higher than those seen in 2005, despite the recent recession, which has seen some of the smaller operations making a loss. Thus, we should take reported lodge profits (Supporting Information Table S1) as a minimum estimate.

On the other hand, as we discuss above, the lodges use some of the TNR to stage hikes (6795 ha, assuming an 800m buffer), although they pay park fees for this (US$ 208,560 in 2005). One could argue that profits and NPV should be normalized by the sum of ecotourism-controlled outside the TNR and ecotourism-used land inside the TNR, which would have the effect of reducing profits per hectare by a divisor of 1.2 ( = (6795+31807)/31807). On balance, the two effects (true profits being roughly double those reported in 2005 but a larger land-area in the denominator) probably more strongly favors our conclusion that ecotourism is the most valuable use of rainforest in Tambopata.

Furthermore, our scenarios in which agriculture replaces ecotourism (Table 1) assume conservatively that, relative to farms near the main roads, transport costs are not higher from ecotourism concession land. This would reduce profits from agriculture.

#### *Discount rate*

Private discount rates can be higher than public ones, such as the 7.35% that we use here. A higher discount rate would make unsustainable logging more privately attractive than ecotourism, since profits from logging are front-loaded. We do not know what private discount rate is appropriate for loggers in Tambopata, but we emphasize that our profit estimates from ecotourism are quite conservative, that concurrent selective logging away from tourist trails is in principle compatible with ecotourism (although not allowed under ecotourism concession rules), that the carbon sequestration market could eventually provide an additional income stream for intact forest, and that we do not contemplate that a single individual is choosing between the alternatives. Most loggers could not possibly run a lodge, and tour operators do not engage in logging in their concessions. Instead, the better way of thinking about this is that tour operators are at least as incentivized by profits to use forest cover to run tours as loggers are to use forest to extract timber. This conflict over land use is adjudicated in the courts and marketplace.

#### *Tax policy*

Legislation to promote business development in Amazonian Peru includes low corporate income and sales taxes for lodges, ranging from 5–10%. In contrast, tax rates on formal-economy ranchers and loggers range from 10–15%. Obviously, a lower tax rate on lodges inflates the private profit-value of ecotourism, but has less effect on producer surplus estimates, as fixed costs include some taxes. In any event, small-scale farmers and informal loggers (who are the ones who engage in unsustainable logging) pay little to no tax, meaning that the main competitor to ecotourism is, effectively, even more favored by the current tax regime.

#### *Carbon*

Another way of calculating the net effect of carbon emissions and sequestration would have been to estimate a revenue stream from private-market carbon sequestration projects and an avoided-damage cost for emissions. However, valuations are highly variable and uncertain, and we chose the simpler like-for-like comparison here. That said, lodges are currently (2009/2010) discussing the possibility of using carbon sequestration funds to acquire new land and to create benefit-sharing packages with neighboring communities.

For lack of data, we did not include carbon and methane generation from agriculture (e.g., animal husbandry and soil changes), but we also did not include any carbon emissions by tourists for their international flights or local food consumption. Our logic is that local food consumption by tourists is a substitute for consumption in their home countries. For international flights, we have no way of calculating how many fewer tourists would enter Peru if the Tambopata rainforest package were not available, given that the primary draw in Peru is the Inca city of Macchu Picchu (only ∼6% of foreign tourists to Peru visit Tambopata). Regardless, the huge amount of sequestered carbon on ecotourism concessions means that one could include all or most of the carbon emitted on international flights and still be left with hundreds of years of carbon-neutral operation.

#### *Socio-cultural effects on the local population*

We have also omitted consideration of the effects on local cultures and norms by ecotourism. This is because the local society is largely the product of recent immigration from the Andes and is therefore in flux anyway and because other global influences have far stronger effects, such as the booming market for gold, which has fomented violent protests by miners demanding access to all land classes [41], and the Interoceanica Highway, which will have a myriad of effects, including the influx of thousands of truckers. Relative to these effects, we are persuaded that ecotourists and ecotourism have had benign and minor effects on local culture, especially since the tourists do not spend time in Puerto Maldonado. Also, rainforest guiding is a preferred employment option amongst youths, local guiding schools and courses have been established, and most guides now originate from Puerto Maldonado.

### Conclusion

The use-value to Peru of ecotourism-controlled land in this portion of Peru is the 25-year NPV of producer surplus: US$ 1,158 ha^−1^. We conclude that ecotourism is the single most valuable use of tropical forest in Tambopata, Peru. Consideration of potential sources of error only reinforces this conclusion, as the true, current profits of ecotourism are probably much higher than those quantified in Supporting Information Table S1. We also find that ecotourism in Tambopata is, at the least, carbon neutral. The surplus of sequestered carbon over that emitted by transport adds to the social value of ecotourism, although it is difficult to value.

Looking backwards, we conclude that the 2002 policy decision to introduce ecotourism and conservation concessions has been justified on narrow economic grounds alone. Forest concessions are the key policy instruments that ‘close the ecotourism-conservation’ loop by increasing the expected return on conservation actions and allowing lodges to continue operations in the face of deforestation pressures [1]. This in turn has allowed the economy to enjoy the highest possible profits and revenues that can be extracted from this area. High profitability also means that ecotourism does not require a favorable tax rate to be viable, which is consistent with recent government proposals to abolish tax subsidies for rainforest firms.

Looking forwards, the high value of ecotourism-controlled land can be used to guide decisions ‘on the margin.’ For now, allowing more lodges to be built or more land to be put into ecotourism concessions, even at the expense of local agriculture, can be justified, as is continuing to protect the local protected areas. In particular, we have identified a vulnerable patch of forest near the city of Puerto Maldonado, known as the “Jorge Chavez-Loero Gap” (Fig. 1), that if protected from further deforestation, will prevent most deforestation from entering the TNR for the next two decades [1]. Because this area is not suitable for ecotourism, there is little individual incentive for lodges to protect the area, and state-led protection is required. In fact, a guard post has recently been installed. Given that deforestation in the TNR will reduce the attractiveness of the forest for tourism, our analysis provides justification for public expenditures to prevent agriculture from entering the Jorge Chavez-Loero gap.

Finally, even though this study has concentrated on financial measures of value, we stress that there are of course other, more difficult-to-quantify reasons to protect forest, including their value as a store of carbon and biodiversity. Thus, looking further to the future, great sources of uncertainty are the prices of gold, agricultural products, and timber. The Interoceanica Highway could potentially result in large price rises for the latter two, as exports to urban Peruvian markets and possibly to other countries becomes feasible. On the other hand, Peruvian producers might find themselves outcompeted by Brazilian agroindustry. Similarly, nominal gold prices are at historical highs, and there is a possibility that more gold miners in the region will find it profitable to move from riverbank placer mines to rainforest, as has occurred in the Huaypetue region of Madre de Dios and in the Jayave and Guacamayo areas between the IOS and the Inambari rivers, all outside the tourism zones [41]. Alluvial gold extraction currently is probably more profitable than tourism, but alluvial gold mining takes places on riverbanks, not in forest, and so are not direct competitors for land. We do not know if any of the rainforest areas used by tourism contain sufficient gold deposits to attract mining, nor whether extraction from rainforest proper is also profitable to the same degree.

In short, ecotourism currently outcompetes alternative uses of forest land in terms of the economic benefits generated from marketable products, but this might not always be the case. In the future, the pre-eminence of ecotourism might only be sustained if ways are found for ecotourist operators to capture the non-market benefits of their operations, perhaps through some form of payment for ecosystem services.

## Methods

### Study area and ecotourism development

The study area is centered on Tambopata, a popular ecotourism destination located in the Department of Madre de Dios, in Amazonian Peru (Fig. 1). In 2008, numbers of tourists and lodges peaked at 52,000 and 37, respectively, but as a consequence of the global economic recession, dropping by approximately 10% in 2009 due to the global recession. Descriptions and histories of the Tambopata tourism sector are in [1], [3], [53]–[55].

The area is dominated by biodiversity-rich lowland tropical forest, much of which is included within a PA complex consisting of the TNR (274,690 ha, established in 2000, parts of which are used for ecotourism and Brazil-nut extraction) and the BSNP (1,091,416 ha, established in 1998, which is largely off limits to people except for a select number of ecotourists, research biologists, and native Ese'eja people). Surrounding these PAs are buffer zones (455,274 ha), extending 2–25 km from the PA boundary northwards as far as the Madre de Dios river, westwards to the IOS highway, and southwards to montane and pre-montane forest ridges. Both PAs abut the Madidi National Park in Bolivia to the east. In this way, they are also vital PAs in the 17-PA network that makes up the Vilcabamba-Amboró Conservation Corridor [56] that stretches from the Vilcabamba area in central Peru to Amboró in central Bolivia. The study area also contains the provincial capital Puerto Maldonado (56,026 inhabitants in 2005), which is served by an international airport and through which virtually all Tambopata's ecotourist visitors transit; 137 rural villages and communities with a combined population of 17,806 inhabitants; and a 230-km section of the IOS highway and associated secondary roads (Fig. 1).

A broad-stroke command-and-control-type EEZ land-use planning strategy was undertaken in Madre de Dios in the late 1990s, with landscape level land-use planning maps published in 2000 [39]. Along with a major change in the wildlife and forestry law [57], [58], the EEZ paved the way for large areas to be leased by the government to private owners as timber, reforestation, Brazil nut, conservation and ecotourism concessions. The EEZ plans are in the process of being updated, as the original plans did not thoroughly take into consideration the paving of the IOS highway or any of the other planned infrastructure projects that are currently in development or under investigation, such as the Inambari dam and hydroelectric power station, located 100km to the west of Tambopata's ecotourism zones, major electricity transmission lines connecting hydroelectric power stations in the south of the country (including Inambari) to major cities in western Brazil, and a transcontinental railway line.

### Ecotourist lodge sample

The owners of twelve ecotourist lodges that had been operating in Tambopata for at least one year on 1^st^ January 2005 were invited by the lead author to provide visitor, land-use, and economic (financial and accountancy) information pertaining to the year 2005, including details of all revenues and expenses. These included (i) two small lodges entirely managed by local families with <1,000 visitors yr^−1^; (ii) two research stations with <1,500 visitors yr^−1^; (iii) three medium-sized lodges with <3,000 visitors yr^−1^; (iv) two medium sized lodges with 3,000–6,000 visitors yr^−1^; and (v) three relatively large lodges with >6,000 visitors yr^−1^. Of these, four lodges are co-owned by partnerships between a Peruvian and an expatriate foreigner under either a formal business agreement or a partnership based on marriage, where in each case the expatriate has been resident in the Peru for at least 10 yrs; two lodges are wholly or partly owned by one set of people; two lodges are the result of partnerships between Peruvian entrepreneurs and local indigenous or mestizo communities; two lodges have been operating since the mid 1970s; and one lodge is part owned by a large Peruvian hotel chain. In 2005, these 12 lodges catered for 35,255 ecotourist visitors, 89% of the total. Our dataset therefore is close to a complete measure of the sector.

### Ecotourism-controlled versus ecotourism-used land

**Figure 3:**
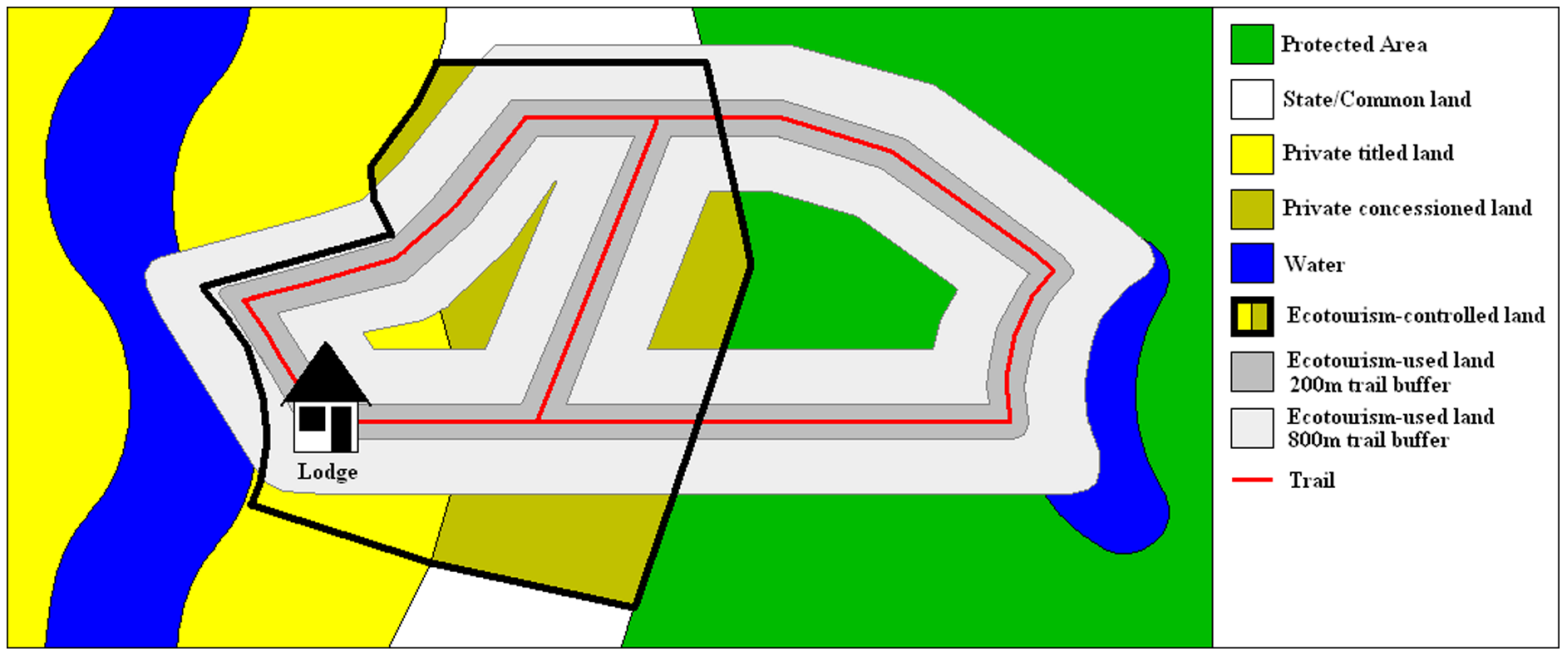
Used versus controlled land. Schematic diagram illustrating the difference between ecotourism-controlled land (area bounded by thick black line), and ecotourism-used land calculated from trail buffers (light and dark gray areas).

All lodges in Tambopata, except for one, are built on private titled land (similar to freehold land in the United Kingdom) that is owned by one or more of their shareholders [3]. The one exception is a lodge located within what is now the TNR but was built prior to the establishment of the Reserve. This lodge was built in 1989 for biological research purposes, but in 1994 the main remit of new investment was ecotourism and under a special lease recognizing prior use rights a more substantial lodge was built. In 2000, the government reformed the wildlife and forestry law [57], [58] and began leasing State-owned land for renewable 40-yr periods to private companies and individuals, beginning with Brazil nut and conservation concessions, followed by timber and reforestation concessions, and finally, in 2004, ecotourism concessions [59]. Lodge owners were quick to lease land in a bid to gain control of strategic access points, intact forest bordering rivers, ox-bow lakes, and clay-licks (*collpas*), which are visually attractive habitats and which naturally concentrate wildlife species that tourists like to observe [1]. Private titled lands and leased concessions owned by ecotourist lodges we term here as *ecotourism-controlled* land, in that lodges control legal access rights (Figs. 2 and 3). The twelve lodges at the center of this case study provided georeferenced maps of the lands they control, which were analyzed using ArcGIS 14.0 (ESRI) software to determine the area of land in each case.

In most cases, however, the land that a lodge controls does not coincide with the areas it actually uses during the provision of services such as guided walks along forest trails or visits to ox-bow lakes and clay-licks. Many of the trails, lakes and clay-licks are located inside the TNR, and access is only possible having paid an entrance fee. The lands either side of the trails within visual and auditory distance we term here as *ecotourism-used* land. The visual distance was estimated to be 200m, corresponding to the maximum distance a person is able to see through the forest such that the immediate visual impression when looking out from a trail is one of being in an intact forest. The auditory distance was calculated at 800m and corresponds to the average distance the lead author was no longer able to hear a working chainsaw whilst standing on a trail in thick forest, as determined using a hand-held Global Positioning System (GPS) unit, and is regarded as sufficient to provide a visitor with the impression that they are immersed in an intact tropical forest ecosystem. Some lodges provided georeferenced maps of the trails they use and the attractions they visit, and for others the lead author collected this information using a GPS. ArcGIS software was used to generate 200m and 800m buffers around the trail systems of lodges, as depicted in Fig. 3, in order to calculate the area of forest used in each case.

### Private benefits of ecotourism: Lodge profits

The twelve participating lodges provided detailed accountancy data for 2005 pertaining to their revenues and expenses (fixed costs, variable costs, and sunk costs), as well as the geographic distribution of costs (i.e. where in Peru the cost transactions took place) and the number of salaried workers (i.e. first order beneficiaries). This permitted a financial analysis of each lodge and the calculation of pre-tax profits (hereafter referred to as profits). Due to the commercially sensitive nature of this information, profits are not described on a per lodge basis, but rather pooled.

Lodge profits were subsequently divided by the amount of *ecotourism-controlled* land, which provides an estimate of the 2005 profit-value of forest land for ecotourism in Tambopata. The profit value was used to calculate the net present profit-value (NPV), assuming a 25-year time horizon and a discount rate of 7.35%, which is the underlying cost of capital in Peru (i.e. the long-term interest rate on sovereign Peruvian debt): NPV_δ = 7.35%, 25 years_.

The NPV of ecotourism land is expressed in two ways. The first is the profit value of land averaged across lodges (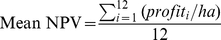), and the other is the profit value of land combined across lodges (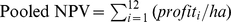). This latter value was calculated by dividing the sum of profits for all twelve lodges by the sum of all the lands either controlled or used by lodges. We do this because there is considerable heterogeneity across lodges, and a combined NPV can give a better picture of the sector.

### Private opportunity costs of ecotourism: Alternative land-use profits

#### *Agriculture, cattle ranching, and unsustainable timber extraction*

To determine the private opportunity costs of ecotourism-controlled lands in 2005, we first undertook a household survey in 2006 to estimate household net economic returns to land (pre-tax profit value of land, 2005 US$ ha^−1^). As with lodge profits, we calculate the NPV_δ = 7.35%, 25 years_.

The study area was first stratified into seven geographical areas chosen to reflect a range of village types (old, new, large, small) and likely variations in household's net returns to land across the landscape (Fig. 2). These included (i) villages located within 20 km of Puerto Maldonado, the principal market town, and accessible by the highway, secondary roads and major rivers; (ii) villages located on or within 15 km of four sections of the Interoceanica Highway from (a) Sudadero to Planchón (30 km section), (b) El Castañal to Laberinto (30 km section), (c) Asociacion Residentes Cusqueños to Union Progreso (30 km section), and (d) Nueva Arequipa to Santa Rosa (30 km section); (iii) villages located along an 80 km section of the Madre de Dios river between Fortuna and San Juan Grande; and finally (iv) villages located on the middle reaches of the Tambopata river, whose lands lie closest to the TNR. Four interview teams, each composed of two local Peruvians, were assembled by the lead author and trained in interview and questionnaire techniques. Members of these teams were chosen based on their prior knowledge of the sample areas, having worked with local farmers, timber extractors, educators, health professionals and government survey teams in these areas in the preceding two years. Involvement of trained Peruvians in this way allowed efficient sampling of households in each geographical area of interest and, we believe, reduced the chances of *outsider* bias, a phenomenon whereby interviewers perceived to be outsiders by the interviewee are treated with a certain degree of mistrust, resulting in unreliable information being collected, particularly when it comes to sensitive economic and livelihood information where trust between interviewer and interviewee is paramount.

The household selection process involved a stratified random sampling design, where villages in each of the seven geographical areas were identified, which included the largest village in each area and up to seven of the smaller ones. Individual households were chosen based on a starting point located near the middle of the village and along one (small villages, <200 people) or two routes (large villages, >200 people) leading off towards the boundary of the village, along which an initial, rapid, door-to-door assessment of households on either side of the route was undertaken to determine the degree to which households were willing to participate in the study. In each case, households were approached by the interview team, and when greeted by a household member, one of the interview team gave a brief explanation of the questionnaire, the approximate time it would take to complete and the type of incentives that a household member could gain by participating, followed by an enquiry as to their willingness to participate. Those households that expressed a willingness to participate were subsequently scheduled for at least one repeat visit during which semi-structured interviews were undertaken. Those households that were empty or did not express a willingness to participate in the study were not subsequently interviewed. No data on the characteristics of these non-participating households were collected, so our study is biased towards households willing to share information.

A total of 209 households participated. Between one and three visits were made to these households, and efforts were made to interview the head of the household in each case. Households provided information on (i) the size and legal status of the land parcels they owned or managed; (ii) the area and annual yields of land under agriculture and fruit production (rice, maize, cassava, bananas, citrus and papaya), animal husbandry (cattle, pigs and chickens), and that used for timber extraction; (iii) the land area in fallow; and (iv) household revenues and expenses (including transportation costs of goods sold at market) from productive activities associated with the growing season of 2006. Incentives were offered to household heads in some cases to encourage them to dedicate the necessary time to the interview, particularly if the information was not possible to obtain during one visit. Seventy percent of households accepted the incentives when offered, which took the form of food parcels or occasionally seed and simple farm implements that were specifically requested by household members. The mean value of the incentive per household was US$4, a little less than the US$5 paid for a typical day of agricultural labor in Tambopata.

Household revenue and expense data associated with productive activities was used to calculate household net income (pre-tax profits) from each activity. Revenues were used to place households into one of 12 land-use categories, based on the activity that generated ≥50% of annual revenues, as a proxy for the degree of household specialization in economic production. With a GPS, the geographic location of each household was determined. This information was manipulated in ArcGIS and overlaid with settlement information, transportation networks (primary and secondary roads, and navigable rivers) and protected areas, and subsequently used to calculate an un-weighted and weighted proxy for the degree of accessibility of each household. The un-weighted proxy consisted of summing the travel distance (km) along all sections of road and rivers used to transport produce from a household to Puerto Maldonado, whilst the weighted proxy corresponds to a more realistic value based on the travel cost of getting produce to market that incorporates the variation in transport costs on the IOS highway, secondary roads, and navigable rivers, as determined directly from data provided by households.

Households provided information on the annual yield of different produce, the income obtained from the sale of produce, and the transportation costs associated with each unit of produce when taken to market. Produce that was consumed by household members or fed to livestock was given a shadow price equal to the income obtained if the produce were sold plus the relevant transportation costs. From this gross income was subtracted the costs of production (i.e. inputs and labor) to provide a value of net income (pre-tax profits) for each produce. Profits were summed across all product lines to arrive at a household's net income for 2006, which was converted to 2005 US$ using the US Bureau of Labor Statistics' consumer price index (CPI) inflation calculator [60].

#### *Timber extraction from ecotourism concessions*

The opportunity costs of intact forest within the concession portions of ecotourism-controlled land lie primarily with the timber resources on these lands, i.e. stumpage value. This logic assumes that if these forests had not been controlled by ecotourist lodges then it is likely the government would have allocated the property rights to timber companies, as indeed it did with the majority of forests in Madre de Dios outside of PAs. To calculate the per-hectare NPV of commercial timber on ecotourism concessions, we first calculated the area of suitable forest in these concessions by superimposing the boundaries of each concession onto a map of intact forest and then extracting the area associated with palm swamps and secondary forest, which do not contain meaningful volumes of commercial timber. The result was a total 16,016 ha of suitable timber forest.

To calculate the per-hectare NPV of commercial timber on this land, we first calculated the area of suitable forest (i.e. disregarding palm swamps and secondary forest, which do not contain meaningful volumes of commercial timber) by superimposing the boundaries of each ecotourism-controlled concession onto a map of intact forest (suitable for timber extraction). The result was a total 16,016 ha of suitable timber forest. We then estimated the mean per-hectare timber or stumpage volume of commercial timber in this forest by analyzing 89 hectares of tree plot data collected at five locations around Tambopata between 1994 and 2007 (Supporting Information Table S2). Only those trees with a diameter breast height (DBH) >50 cm and listed by the state forestry bureau (Instituto Nacional de Recursos Naturales, INRENA) in 2005 as being of commercial interest were included in the dataset (Supporting Information Table S3). The stumpage volume, in board feet (bf), of each of 702 trees found in these plots was calculated using equation 1.
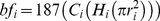
(1)where *bf_i_* is the volume (board feet) of commercial timber in the *ith* tree; r_i_ is the radius in meters of the *ith* tree (i.e. *dbh_i_*/2); *H_i_* is the commercial height in meters of the *ith* tree; C_i_ is a measure of the cylindrical uniformity of the *ith* tree and can be described either as good (0.65), normal (0.60), or poor (0.50); and the value 187, which is the product of 220×0.85, corresponding to a combined conversion and correction factor that first converts volume from m^3^ to bf (where 1 m^3^ = 220 bf) and subsequently corrects the volume by a factor of 0.85 to take account of expected wastage during felling, on-site processing of felled trunks into boards using chainsaws, and imperfections in the timber.

In twelve out of the 89 ha, no data were collected on the commercial height (i.e. height to the first major branch) and degree of cylindrical uniformity of trees, variables that are required to determine a tree's volume. Therefore, for trees in these plots, we used the mean commercial height (12.57 m) and the median cylindrical uniformity category (normal, 0.60) of the 615 trees from the 77 ha for which such data did exist. These 77 ha had been inventoried by professional forest engineers following guidelines laid down by INRENA for the purpose of accurately estimating commercial timber volumes and thus for granting timber extraction rights on both timber and reforestation concessions. Reforestation concessions permit timber extraction after government approval of management plans, which include estimates of maximum annual extraction volume for each approved segment of a concession. They tend to differ from normal timber concessions only in size, reforestation concessions being significantly smaller.

The *sustainable* annual extraction rate of timber from suitable timber forest on ecotourism-controlled concessions was calculated in the same way as that for two existing and operational reforestation concessions that permit selective extraction, as described in their management plans, copies of which were obtained from the NGO ProNaturaleza [61], [62]. It was assumed that the area of suitable timber forest on each of five ecotourism concessions could be managed as an independent reforestation concession. Each of these was subsequently divided into five equal blocks following government regulations. These regulations state that timber extraction is permitted in a block during four consecutive years before extraction must move on to another block, and that only 50% of the commercial timber in a block is allowed to be extracted during the first cutting cycle of 20 years. This allows for a second cutting cycle within each block after 20 years for a total combined managed cutting cycle of 40 years - the legal duration of a reforestation concession contract in Peru. The authors have deemed this level of extraction to be sustainable. With 2,380 bf representing half the volume of commercial timber on a typical hectare of suitable forest in Tambopata, the mean extraction intensity in each ecotourism concession was calculated at 381,251±518,386 bf yr^−1^ for a total extraction of 1,906,256 bf yr^−1^, equivalent to only 0.03% of the 2004 extraction intensity in Madre de Dios as a whole [40]. We calculate profits from *unsustainable* timber extraction on ecotourism concessions by extracting and selling all timber on a concession evenly over a short five-year period. The potential increased supply of timber from these concessions would likely, therefore, not have had a price-dampening effect on the gate price of timber (as calculated below).

The concession-gate price of extracted timber in Tambopata is governed firstly by whether the tree species it comes from is slow-growing and produces high quality hardwood (known locally as *madera dura*) or is a fast-growing, lower quality softwood (*madera corriente*). During the household surveys, we collected data on 130 timber transactions, 19 for *madera dura* (total volume of sales: 98,200 bf) and 111 for *madera corriente* (total volume of sales: 465,750 bf). The mean price (±95% confidence interval) in 2006, expressed in constant 2005US$, for hardwood was US$0.43±0.07 bf^−1^ (Peruvian Nuevo Soles S/.1.45±0.24 bf^−1^) and for softwood was US$0.18±0.01 bf^−1^ (S/.0.64±0.03 bf^−1^) (See Supporting Information Table S3 for a list of the commercial timber species and prices analyzed). These prices were very similar to those reported by others [63] and were thus applied to the corresponding volume of timber of each wood type at the five tree inventory sites to calculate the mean revenue, under the assumption that all the commercial timber at each site were cut down and sold, following the rules and regulations set out by government for reforestation and timber concessions. The mean revenue per board foot was found to be US$0.226 bf^−1^. In turn, the average annual cost of extracting a typical board foot of timber from a concession was determined using cost data from the budgets of the aforementioned management plans of reforestation concessions obtained from ProNaturaleza (Supporting Information Table S4). These costs included sunk capital expenses such as the purchase of chainsaws, planking equipment and spare parts, whose average annual cost over twenty years at 2005 prices was calculated at US$460 yr^−1^. The mean cost (Free on Board, FOB) of timber extraction in this case was calculated to be US$0.112 bf^−1^, a figure similar to that observed in the native community of Bélgica [64]. Thus, the pre-tax profit value of a typical board foot of sawn timber placed at the concession gate in Tambopata was estimated to be US$0.114 bf^−1^ (i.e. US$0.226–US$0.112). This value is an underestimate to some degree, as it does not include any multipliers from further processing of timber into finished consumer products, although most of which occurs outside of Tambopata. On the other hand, we are not counting the multiplier effect of ecotourism spending either.

#### *Sustainable Brazil nut extraction*

Other than timber, the next most likely alternative extractive product from intact forest on ecotourism-controlled concessions is Brazil nuts (*Bertholletia excelsa*), a well known non-timber forest product that is native to the forests of Tambopata and which can occur in high densities [65], [66]. Brazil nut concessions across Madre de Dios, both within and outside of PAs, were formalized in 2000–2004. By 2005, a total of 958 Brazil nut concessions had been authorized for renewable 40-yr periods across Madre de Dios. The vast majority of these were granted to locals with historical rights to the resource, though some were purchased by lodge owners in order to control access to them [1].

To determine the opportunity costs associated by not commercially harvesting Brazil nuts on ecotourism-controlled concessions, we first analyzed area and productivity data provided by INRENA for three consecutive years (2004, 2005, 2006) pertaining to 67 Brazil nut concessions located within the TNR/BSNP protected area complex and associated buffer zones. These concessions account for 69% of all concessions in Tambopata and cover 59,780 ha. After performing a natural log (ln) transformation to smooth out the variance, the mean annual production (kg ha^−1^) across concessions varied at most marginally significantly over the three year period for which data was available (2004: Mean 7.65+/−0.63 SE, 2005: 7.26+/−0.59, 2006: 9.75+/−0.83; ANOVA, n = 201, F_2,198_ = 2.638, P = 0.074). We therefore pooled productivity data across 67 concessions for all three years to estimate a mean productivity of 8.22 kg ha^−1^ yr^−1^±0.41 SE. Economic data on annual revenues, costs, and thus pre-tax profits was available for only one of the 67 concessions. Therefore, data for these economic variables were gathered from separate studies of 26 Brazil nut concessions located outside of the TNR, BSNP and buffer zone, for a total of 27 concessions (Supporting Information Table S5). These included one native community (Comunidad Nativa de Pariamanu) that extracted Brazil nuts from its communal lands and is treated here as a concession (although long-term rights to the resource are safeguarded, unlike concessions which can be repealed by the government). In 24 of these concessions, the owners were interviewed between 2005 and 2008 (C. Kirkby = 2, A. Dueñas-Dueñas = 2, L.M. Velarde-Andrade = 20) and provided area, productivity and economic (revenues, costs, profits) data for their concessions, whilst similar data on a further three concessions was gleaned from the grey literature in Peru [67]. With this information, and converting economic values to constant 2005US$, it was possible to estimate the pre-tax profit value and thus the NPV_δ = 7.35%, 25 years_ of land (US$ ha^−1^) associated with each of the 67 Brazil nut concessions of interest.

### Social costs of ecotourism: Carbon emissions from tourism

Ecotourists travel to and from a lodge in Tambopata by plane, bus and boat. We calculated travel-related carbon emissions by first calculating the domestic return flight distance (628 km) between the city of Cusco (the principal tourist destination in Peru) and Puerto Maldonado (gateway city to Tambopata), the common route that all ecotourists to Tambopata take. By restricting our analysis to this route, we are assuming that tourists primarily choose to travel to Peru from their home countries for other attractions, such as the Inca citadel of Macchu Picchu. Rainforest tours are typically sold as optional extensions to package tours of Peru. We thus multiplied the Cusco-Puerto Maldonado return flight distance by the number of visitors to each lodge in 2005 to obtain the number of person kilometers (pkm) travelled for each lodge. We then took the mean of five estimates of the weight of carbon burned per pkm (kg C pkm^−1^) on a domestic flight, based on data from Miyoshi [68], and multiplied this by the pkm travelled for each lodge to give the total flight emissions per lodge (measured in tons of carbon, tC). Data on fossil fuel consumption by the 12 lodges in 2005, which was assumed to be primarily used for ferrying visitors by bus and boat from the Puerto Maldonado airport to the lodges and back, was used in conjunction with US Environmental Protection Agency estimates of the carbon content of gasoline (2.421 kg C gallon^−1^, [69]) to estimate terrestrial emissions per lodge. Total emissions per lodge for 2005 were then calculated by adding their respective flight and terrestrial emissions.

### Social benefits of ecotourism: Avoided carbon emissions

The intact tropical forests of Tambopata, including boundary regions of the TNR including most if not all of the ecotourism lands within the TNR and its buffer zone, are threatened with deforestation as a result of high human population growth rates and the concomitant expansion of activities such as agriculture, cattle ranching, timber extraction and placer gold mining, particularly in areas along secondary roads, navigable rivers, on the periphery of Puerto Maldonado, and within 30 km of the IOS highway. Nevertheless, the profit motive and a desire to conserve wild nature amongst lodge owners has led them not only to increase over time the area of forested lands coming under their control but also to enact a plethora of conservation actions to maintain forest cover on these lands [1].

Elsewhere, deforestation scenarios at the level of Madre de Dios and Tambopata, respectively, were modeled for the period 2005–2035 [1], [70], using *Dinamica EGO* software, a stochastic cellular automata model that has been successfully used in the past to model deforestation scenarios in the Amazon [71]–[73]. The two deforestation scenarios tested for Tambopata were a business-as-usual (BAU) scenario, where the historic population-based deforestation rate was projected into the future (taking into account expected population growth in urban and rural areas), and an ecotourism-led conservation (ECO) scenario, where the ecotourism-controlled lands in Tambopata are actively protected from deforestation.

The difference in deforestation between the scenarios and within the ecotourism-controlled lands is taken to be the reduction in deforestation attributable to ecotourism. This reduction in deforestation can be measured in terms of tons of carbon per annum not emitted into the atmosphere and is thus a social benefit from the perspective of mitigating climate change. We therefore took the annual change in deforestation area due to ecotourism and multiplied it by the expected change in above-ground carbon content between primary tropical forest and a tropical agricultural landscape typical of this region of the Amazon. The above-ground carbon content of primary tropical forest in Tambopata is 172 tC ha^−1^ [74], and a typical mixed-agriculture landscape stores 15 tC ha^−1^ [75], assuming 50% of above-ground biomass is carbon, for a difference of 157 tC ha^−1^.

### Producer surplus values of ecotourism and alternative activities

We calculated the 2005 producer surplus (PS) of each lodge. While profit measures the private benefits to ecotourism operators of their activities, economists usually measure the social benefit of those activities through the broader measure known as Producer Surplus (PS), which is calculated as PS = profits+fixed costs [76], where fixed costs are the minimum expenses required to maintain operations and, in most lodges, are separated out in the accounting. Examples include salaries, benefits, food, and transport for year-round staff (thus excluding seasonal employees such as guides), office rentals and expenses, maintenance, and taxes. For example, over 2005, one lodge classified $188,530 as fixed costs, or $15,710 per month. This corresponds closely to the mean of the total expenses incurred during the four lowest-volume months, January to April ($14,145 per month).

Even this broader measure does not consider the PS accruing to the two airlines that service the route between Cusco and Puerto Maldonado that virtually all ecotourist visitors to Tambopata take. In the absence of data on fixed costs for the airlines, the PS value was assumed conservatively to equal the profits of the dominant airline (Lan Peru) for the route [1].

We then used the sum of lodge PS and airline profits to recalculate the NPV per hectare used and controlled, following the methods in ***Private benefits of ecotourism: Lodge profits*** above.

We also calculated the 2005 producer surplus (PS) of the various alternative activities. Examples of fixed costs include the value of (i) regular land clearance/weeding (chainsaw, petrol, labor, food consumed by labor); (ii) food consumed by landowner or caretaker whilst on the land (usually derived from harvested crops), plus any stipends paid to a caretaker; (iii) purchase or rental, plus maintenance, of implements and machinery, (iv) cost of monthly veterinary visits (but not the treatments, which are variable costs), and so forth.

An intuitive way of thinking about PS and why it is a measure of the social value of a business is that profits plus fixed costs is the maximum amount that a state could tax a business before the business would choose to stop operations. By definition, in the short term, a business must pay its fixed costs regardless of its profitability (e.g., a fixed-term lease on an office). Thus, even if a business were to have all profits removed by tax, it would still be willing to continue operations if the margins on sales were sufficient to cover fixed costs. If profits plus fixed costs were removed by tax, then the business would choose to cease operations. Thus, PS represents the maximum amount of money that *could* be taxed and distributed to society by a notional central planner, and therefore measures the social value of a business. We emphasize that we make no normative judgments here about how much tax *should* be levied on private businesses.

Alternatively, one can imagine that an ecotourism business is prevented from operating and earning profits (such as agriculture taking over former ecotourism land). The ecotourism business suffers a loss of welfare, and to be compensated fully, it would require a payment of both its lost profits and its fixed costs, the latter of which, by definition, must be paid out whether the business is producing or not. This compensation is the welfare value of the firm. Note that all welfare is individual, not societal, so that Producer Surplus is therefore the full compensation value of the ecotourism business as well as the social value of that business. This value is different from the economic impact of a business, which is some measure of the revenues plus multiplier effects of spending.

## Supporting Information

Table S1 Valuation of land use in Tambopata, Peru. The total and mean (±95% confidence interval) land area, profits (pre-tax), profit-value of land (ha^−1^), net present value (NPV) of profit-value, producer surplus (PS), and NPV of PS of ecotourism lands and alternative activities (annual crops, fruit, cattle, timber, Brazil nuts). All monetary values are expressed in 2005 US$. Ecotourism profits are from 2005, and alternative activities data are from 2006. Columns headed “Pooled” correspond to summed data across N samples, whilst those headed “Mean” correspond to the average across N samples. NPV was calculated based on a 25-year time horizon and a discount rate of 7.35%.
(0.14 MB DOC)

Table S2 Characteristics of the five inventoried forest areas in Tambopata used to calculate mean commercial timber volume. TF, Terra firme forest; FF, Floodplain forest. The list of commercial timber species used was based on primary information provided by the Forestry and Fauna Department (IFFS) of INRENA in Puerto Maldonado and is also detailed by León-Cornejo and Mego-Canta [1].
(0.06 MB DOC)

Table S3 Commercial timber species and prices per board foot (bf), taken from actual transactions information collected during household surveys, used to calculate the board foot (bf) value of each species. The list of commercial timber species used was based on primary information provided by the Forestry and Fauna Department (IFFS) of INRENA in Puerto Maldonado and is also detailed by León-Cornejo and Mego-Canta [1].
(0.11 MB DOC)

Table S4 The mean annual cost (per board foot) associated with managing and extracting timber from reforestation concessions over a 20-yr period (2005–2024). The values do not include (i) payment of extraction rights (US$0.0027 bf^−1^) and sales taxes (19%) to the government; (ii) depreciation of capital goods (chainsaws, etc.); and (iii) amortization of loans. Based on data from management plans for two reforestation concessions prepared by ProNaturaleza [1], [2].
(0.05 MB DOC)

Table S5 Area and productivity characteristics of the sampled Brazil nut concessions in Tambopata, associated with the Tambopata National Reserve (TNR) and Bahuaja-Sonene National Park (BSNP), and the Economic sample for which financial information on revenues, costs and pre-tax profits was available.
(0.04 MB DOC)

## References

[1] Kirkby C, Day B, Turner K, Soares-Filho BS, Oliveira H (in press). Closing the ecotourism-conservation loop in the Peruvian Amazon.. Environmental Conservation.

[2] Ceballos-Lascurain H (1987). The future of ecotourism.. Mexico Journal.

[3] Yu DW, Hendrickson T, Castillo A (1997). Ecotourism and conservation in Amazonian Peru: short-term and long-term challenges.. Environmental Conservation.

[4] Mackoy RD, Osland GE (2004). Lodge Selection and Satisfaction: Attributes valued by ecotourists.. The Journal of Tourism Studies.

[5] Balmford A, Beresford J, Green J, Naidoo R, Walpole M (2009). A global perspective on trends in nature tourism.. PLoS Biol.

[6] Gossling S (1999). Ecotourism: a means to safeguard biodiversity and ecosystem functions?. Ecological Economics.

[7] Doan TM (2000). The Effects of Ecotourism in Developing Nations: An Analysis of Case Studies.. Journal of Sustainable Tourism.

[8] Honey M (1999). Ecotourism and Sustainable Development: Who Owns Paradise?.

[9] Wunder S (2000). Ecotourism and economic incentives - an empirical approach.. Ecological Economics.

[10] Stronza A (2000). Because it is ours: community-based ecotourism in the Peruvian Amazon [PhD thesis].

[11] Alpizar F (2006). The pricing of protected areas in nature-based tourism: A local perspective.. Ecological Economics.

[12] Brown CR (2001). Visitor Use Fees in Protected Areas: Synthesis of the North American, Costa Rican and Belizean Experience.

[13] Drumm A, Moore A, Soles A, Patterson C, Terborgh JE (2004). Ecotourism Development: A Manual for Conser vation Planners and Managers.

[14] Wunder S (1999). Promoting Forest Conservation through Ecotourism Income? A case study from the Ecuadorian Amazon region.

[15] Walpole MJ, Thouless C, Rosie Woodroffe, Simon Thirgood, Rabinowitz A (2005). Increasing the value of wildlife through non-consumptive use? Deconstructing the myths of ecotourism and community-based tourism in the tropics.. People and Wildlife: Conflict or Coexistence.

[16] Barany ME, Hammett AL, Shillington LJ, Murphy BR (2001). The Role of Private Wildlife Reserves in Nicaragua' s Emerging Ecotourism Industry.. Journal of Sustainable Tourism.

[17] Damania R, Hatch J (2005). Protecting Eden: markets or government?. Ecological Economics.

[18] Salafsky N, Cauley H, Balachander G, Cordes B, Parks J (2001). A systematic test of an enterprise strategy for community-based biodiversity conservation.. Conservation Biology.

[19] Kiss A (2004). Is community-based ecotourism a good use of biodiversity conservation funds?. Trends in Ecology & Evolution.

[20] Pearce D (2007). Do we really care about biodiversity?. Environmental Resource Economics.

[21] Ohl-Schacherer J, Mannigel E, Kirkby C, Shepard-Jr. G, Yu DW (2008). Indigenous ecotourism in the Amazon: a case study of ‘Casa Matsiguenka’ in Manu National Park, Peru.. Environmental Conservation.

[22] Blom A (2000). The Monetary Impact of Tourism on Protected Area Management and the Local Economy in Dzanga-Sangha (Central African Republic).. Journal of Sustainable Tourism.

[23] Reed SE, Merenlender AM (2008). Quiet, Nonconsumptive Recreation Reduces Protected Area Effectiveness.. Conservation Letters.

[24] Karp DS, Root TL (2009). Sound the stressor: how Hoatzins (Opisthocomus hoazin) react to ecotourist conversation.

[25] Griffiths M, van Schaik CP (1993). The impact of human traffic on the abundance and activity periods of Sumatran rain forest wildlife.. Conservation Biology.

[26] Wearing S, Neil J (1999). Ecotourism: Impacts, Potentials and Possibilities.

[27] Wight P (1993). Ecotourism: Ethics or Eco-Sell?. Journal of Travel Research.

[28] Wunder S (2001). Deforestation and economics in Ecuador: A synthesis.

[29] Taylor JE, Hardner J, Stewart M (2006). Ecotourism and Economic Growth in the Galapagos: An Island Economy-wide Analysis.

[30] Drumm A (1991). An integrated impact assessment of nature tourism in Ecuador's Amazon region.

[31] Malky-Harb A, Pastor-Saavedra C, Limaco-Navi A, Mamani-Capiona G, Limaco-Navi Z (2007). El efecto Chalalan: Un ejercicio de valorizacion economica para una empresa comunitaria.

[32] Kirkby C, Yu DW, Ghazoul J, Sheil D (2010). Ecotourism case study.. Introduction to Tropical Rain Forest Ecology and Management.

[33] Chomitz KM, Alger K, Thomas TS, Orlando H, Nova PV (2005). Opportunity costs of conservation in a biodiversity hotspot: the case of southern Bahia.. Environment and Development Economics.

[34] Norton-Griffiths M, Southey C (1995). The opportunity costs of biodiversity conservation in Kenya.. Ecological Economics.

[35] Yu DW, Levi T, Shepard GH (in press). Conservation in low-governance environments.. Biotropica.

[36] Theobald DM, Spies T, Kline J, Maxwell B, Hobbs NT (2005). Ecological support for rural land-use planning.. Ecological Applications.

[37] Lier HNv (1998). The role of land use planning in sustainable rural systems.. Landscape and Urban Planning.

[38] Etter A (1994). Caracterización ecológica general y de la intervención humana en la amazonia colombiana. Zonificación ecológica-económica: Instrumento para la conservación y el desarrollo sostenible de los recursos de la Amazonía.

[39] CTAR, IIAP (2000). Zonificación ecológica, económica de la Región de Madre de Dios.

[40] GOREMAD (2005). Plan estrategico regional del sector agrario, Region Madre de Dios.

[41] Yu DW, Levi T, Shepard GH (2010). Conservation in low-governance environments.. Biotropica.

[42] Gavin MC, Anderson GJ (2007). Socioeconomic predictors of forest use values in the Peruvian Amazon: A potential tool for biodiversity conservation.. Ecological Economics.

[43] Sheil D, Wunder S (2002). The Value of Tropical Forest to Local Communities: Complications, Caveats, and Cautions.. Conservation Ecology.

[44] Balmford A, Bruner A, Cooper P, Costanza R, Farber S (2002). Economic Reasons for Conserving Wild Nature.. Science.

[45] Batagoda BMS, Turner RK, Tinch R, Brown K (2000). Towards policy relevant ecosystem services and natural capital values: rainforest non-timber products.

[46] Naidoo R, Adamowicz WL (2005). Economic benefits of biodiversity exceed costs of conservation at an African rainforest reserve.. PNAS.

[47] Pearce D (2001). How valuable are the tropical forests? Demonstrating and capturing economic value as a means of addressing the causes of deforestation..

[48] Kvist LP, Andersen M, Hesselsøe M, Vanclay JK (1995). Estimating use-values and relative importance of Amazonian flood plain trees and forests to local inhabitants.. Commonwealth Forestry Review.

[49] Torras M (2000). The total economic value of Amazonian deforestation, 1978–1993.. Ecological Economics.

[50] Ricketts TH, Daily GC, Ehrlich PR, Michener CD (2004). Economic value of tropical forest to coffee production.. PNAS.

[51] Menkhaus S, Lober DJ (1996). International Ecotourism and the Valuation of Tropical Rainforests in Costa Rica.. Journal of Environmental Management.

[52] Barnes JI, de Jager JLV (1995). Economic and financial incentives for wildlife use on private land in Namibia and the implications for policy.

[53] Groom MJ, Podolsky RD, Munn CA, Robinson JG, Redford KH (1991). Tourism as a sustainable use of wildlife: A case study of Madre de Dios, southeastern Peru.. Neotropical Wildlife Use and Conservation.

[54] Kirkby CA, Doan TM, Lloyd H, Cornejo Farfán A, Arizábal-Arriaga W (2000). Tourism development and the status of Neotropical lowland wildlife in Tamboapta, South-eastern Peru: Recommendations for tourism and conservation.

[55] Duellman WE (2005). Cusco Amazonico: The lives of amphibians and reptiles in an Amazonian rainforest.

[56] CEPF (2005). Tropical Andes Hotspot: Vilcabamba-Amboró Conservation Corridor..

[57] Anon (2000). Ley (No. 27308) Forestal y de Fauna Silvestre..

[58] Anon (2001). Reglamento (D.S. 014-2001-AG) de la Ley Forestal y de Fauna Silvestre..

[59] SPDA (2007). Iniciativa para la conservacion privada y comunal, Boletin Informativo No. 3.

[60] BLS (2009). Inflation Calculator.

[61] ProNaturaleza (2005a). Plan General de Establecimiento y Manejo Forestal de la Concesion de Reforestacion del Sr. Juan Velasquez Cabrera.

[62] ProNaturaleza (2005b). Plan General de Establecimiento y Manejo Forestal de la Concesion de Reforestacion del Sr. Ciro Alagón Huamaní.

[63] Torres-Padilla J (2006). Plan de negocios 2006 para la Asociacion de concesionarios de reforestacion del sector La Pampa, Madre de Dios, Peru.

[64] Canchaya-Toledo JL, Leon-Toro J (2006). Perfil economico de la Comunidad Nativa de Belgica, Madre de Dios, Peru..

[65] Escobal J, Aldana U (2003). Are Nontimber Forest Products the Antidote to Rainforest Degradation? Brazil Nut Extraction in Madre De Dios, Peru.. World Development.

[66] Kainer KA, Wadt LHO, Staudhammer CL (2007). Explaining variation in Brazil nut fruit production.. Forest Ecology and Management.

[67] Arana-Cardo A, Sequeira V, Torres-Padilla J (2002). Mejoramiento del sistema de cosecha de castaña (*Bertholletia excelsa*) en Madre de Dios y sus impactos en la economia del productor castañero.

[68] Miyoshi C (2008). Present carbon dioxide emission levels in the air transport market..

[69] EPA (2005). Emission Facts: Average Carbon Dioxide Emissions Resulting from Gasoline and Diesel Fuel.

[70] Giudice R (2010). Conservation in southeastern Peruvian Amazon: two approaches.

[71] Soares-Filho B, Alencar A, Nepstad D, Cerqueira G, Diaz MCV (2004). Simulating the response of land-cover changes to road paving and governance along a Major Amazon Highway: the Santarem–Cuiaba Corridor.. Global Change Biology.

[72] Soares-Filho B, Cerqueira GC, Pennachin CL (2002). DINAMICA—a stochastic cellular automata model designed to simulate the landscape dynamics in an Amazonian colonization frontier.. Ecological Modelling.

[73] Soares-Filho B, Nepstad D, Curran L, Cerqueira G, Garcia R (2006). Modelling conservation in the Amazon Basin.. Nature.

[74] Winrock (2006). Carbon Storage in the Los Amigos Conservation Concession, Madre de Dios, Peru.

[75] Fearnside PM (1996). Amazonian deforestation and global warming: carbon stocks in vegetation replacing.. Brazil's Amazon forest Forest Ecology and Management.

[76] Just RE, Hueth DL, Schmitz A (2004). The welfare economics of public policy: a practical approach to project and policy evaluation.

[77] Muchagata M, Brown K (2003). Cows, colonists and trees: rethinking cattle and environmental degradation in Brazilian Amazonia.. Agricultural Systems.

