# Supplementary material for: The Market Triumph of Ecotourism: An Economic Investigation of the Private and Social Benefits of Competing Land Uses in the Peruvian Amazon
Source: PLoS One. 2010 Sep 29;5(9):e13015. doi: 10.1371/journal.pone.0013015 (PMC2947509; doi:10.1371/journal.pone.0013015)
Supplement: Table S1 — Valuation of land use in Tambopata, Peru. The total and mean (±95% confidence interval) land area, profits (pre-tax), profit-value of land (ha−1), net present value (NPV) of profit-value, producer surplus (PS), and NPV of PS of ecotourism lands and alternative activities (annual crops, fruit, cattle, timber, Brazil nuts). All monetary values are expressed in 2005 US$. Ecotourism profits are from 2005, and alternative activities data are from 2006. Columns headed “Pooled” correspond to summed data across N samples, whilst those headed “Mean” correspond to the average across N samples. NPV was calculated based on a 25-year time horizon and a discount rate of 7.35%. (0.14 MB DOC) [file pone.0013015.s001.doc]

**The Market Triumph of Ecotourism: An Economic Investigation of The Private and Social Benefits of Competing Land Uses in the Peruvian Amazon: Supporting Information S1**

Christopher A. Kirkby^1,2,3^, Renzo Giudice-Granados^2^, Brett Day^3^, Kerry Turner^3^, Luz Marina Velarde-Andrade^4^ Agusto Dueñas-Dueñas^5^, Juan Carlos Lara-Rivas^6^ and Douglas W. Yu^1,2,*^

^1^ Ecology, Conservation, and Environment Center (ECEC), State Key Laboratory of Genetic Resources and Evolution, Kunming Institute of Zoology, Chinese Academy of Science, Kunming, Yunnan, China

^2^ Center for Ecology, Evolution and Conservation (CEEC), School of Biological Sciences, University of East Anglia, Norwich, Norfolk, UK

^3^ Center for Social and Economic Research on the Global Environment (CSERGE), School of Environmental Sciences, University of East Anglia, Norwich, Norfolk, UK

^4^ Conservación Ambiental y Desarrollo en el Perú (CAMDE-PERU), Puerto Maldonado, Madre de Dios, Peru

^5^ Cooperazione e Sviluppo (CESVI), Puerto Maldonado, Madre de Dios, Peru.

^6^ Universidad Nacional San Antonio Abad del Cusco (UNSAAC), Puerto Maldonado, Madre de Dios, Peru

* Corresponding author: dougwyu@gmail.com

**Acronyms**

BSNP: Bahuaja-Sonene National Park

PS: producer surplus

INRENA: Instituto Nacional de Recursos Naturales

DBH: diameter at breast height

BAU: business as usual DINAMICA scenario

ECO: ecotourism-led conservation DINAMICA scenario

GPS: geographical positioning system

EEZ: ecological and economic zoning

IOS: Interoceánica Sur Highway

PA: protected areas

NPV: net present value

SPDA: Sociedad Peruana de Derecho Ambiental

TNR: Tambopata National Reserve

**Table 1.** Valuation of land use in Tambopata, Peru. The total and mean (± 95% confidence interval) land area, profits (pre-tax), profit-value of land (ha^-1^), net present value (NPV) of profit-value, producer surplus (PS), and NPV of PS of ecotourism lands and alternative activities (annual crops, fruit, cattle, timber, Brazil nuts). All monetary values are expressed in 2005 US$. Ecotourism profits are from 2005, and alternative activities data are from 2006. Columns headed “Pooled” correspond to summed data across N samples, whilst those headed “Mean” correspond to the average across N samples. NPV was calculated based on a 25-year time horizon and a discount rate of 7.35%.

|  | **N** | Land area | | | 2005-6 Profits | | | 2005-6 Profit-value of land | | | NPV (Profit-value of land) | | Producer Surplus-value of land | | | NPV (PS-value of land) | |
| --- | --- | --- | --- | --- | --- | --- | --- | --- | --- | --- | --- | --- | --- | --- | --- | --- | --- |
|  |  | Pooled | Mean | 95% CI | Pooled | Mean | 95% CI | Pooled | Mean | 95% CI | Pooled | Mean | Pooled | Mean | 95% CI | Pooled | Mean |
|  |  | ha | ha |  | US$ | US$ |  | US$ ha^-1^ | US$ ha^-1^ |  | US$ ha^-1^ | US$ ha^-1^ | US$ ha^-1^ | US$ ha^-1^ |  | US$ ha^-1^ | US$ ha^-1^ |
| **Private benefits of ecotourism** |  |  |  |  |  |  |  |  |  |  |  |  |  |  |  |  |  |
| Ecotourism-controlled land | 12 | 31,807 | 2,651 | 3,350 | 1,238,002 | 103,167 | 58,718 | 39 | 257 | 178 | 472 | 3117 | 96 | 707 | 512 | 1,158 | 8,575 |
| Ecotourism-used land |  |  |  |  |  |  |  |  |  |  |  |  |  |  |  |  |  |
| 200m buffer | 12 | 6,611 | 551 | 132 |  |  |  |  |  |  |  |  |  |  |  |  |  |
| 800m buffer | 12 | 21,160 | 1,763 | 387 |  |  |  |  |  |  |  |  |  |  |  |  |  |
|  |  |  |  |  |  |  |  |  |  |  |  |  |  |  |  |  |  |
| **Private (opportunity) costs of ecotourism** |  |  |  |  |  |  |  |  |  |  |  |  |  |  |  |  |  |
| **On titled land** |  |  |  |  |  |  |  |  |  |  |  |  |  |  |  |  |  |
| Crop and fruit specialists ^a^ |  |  |  |  |  |  |  |  |  |  |  |  |  |  |  |  |  |
| Rice specialists | 60 | 2,449 | 40.8 | 4.6 | 40,235 | 671 | 208 | 16 | 18 | 6 | 199 | 217 | 29 | 31 | 8 | 347 | 375 |
| Maize specialists | 10 | 475 | 47.5 | 25.8 | 14,153 | 1,415 | 1,515 | 30 | 50 | 61 | 361 | 601 | 47 | 76 | 76 | 564 | 919 |
| Cassava specialists | 2 | 143 | 71.5 | 20.6 | 25 | 13 | 231 | 0 | 0 | 3 | 2 | 5 | 1 | 1 | 2 | 13 | 15 |
| Bananas specialists | 5 | 158 | 31.7 | 19.9 | 1,640 | 328 | 251 | 10 | 8 | 4 | 126 | 97 | 15 | 21 | 16 | 187 | 249 |
| Papaya specialists | 1 | 30 | 30 | 0 | 76 | 76 | 0 | 3 | 3 | - | 30 | 30 | 3 | 3 | 0 | 38 | 38 |
| Citrus specialists | 1 | 39 | 39 | 0 | 1,483 | 1,483 | 0 | 38 | 38 | - | 461 | 461 | 68 | 68 | 0 | 826 | 826 |
| Animal specialists ^a^ |  |  |  |  |  |  |  |  |  |  |  |  |  |  |  |  |  |
| Cattle specialists | 19 | 1,417 | 75 | 16.5 | 49,959 | 2,629 | 684 | 35 | 42 | 15 | 428 | 503 | 58 | 66 | 17 | 707 | 803 |
| Unsustainable ^b^ | 4 | 263 | 66 | 35 | 9,946 | 2,486 | 1,650 | 38 | 62 | 59 | 316 | 522 | 48 | 77 | 69 | 587 | 932 |
| Sustainable ^c^ | 15 | 1,154 | 77 | 19.1 | 40,013 | 2,668 | 778 | 35 | 36 | 11 | 421 | 435 | 61 | 63 | 14 | 734 | 768 |
| Chicken specialists | 11 | 481 | 43.7 | 20.3 | 11,857 | 1,078 | 1,417 | 25 | 40 | 42 | 299 | 481 | 35 | 57 | 56 | 426 | 690 |
| Pig specialists | 3 | 50 | 16.7 | 23 | 2,343 | 781 | 1,015 | 47 | 46 | 26 | 569 | 555 | 67 | 65 | 48 | 806 | 785 |
| Generalists ^e^ | 59 | 3,147 | 53 | 7.1 | 82,831 | 1,404 | 321 | 26 | 34 | 9 | 319 | 406 | 44 | 53 | 12 | 533 | 646 |
| Timber specialists (Unsustainable) ^a^ | 29 | 1,862 | 64 | 13 | 64,548 | 2,226 | 1,178 | 35 | 44 | 21 | 421 | 534 | 59 | 75 | 26 | 711 | 911 |
| All households | 200 | 10,251 | 51 | 4.2 | 269,148 | 1,346 | 255 | 26 | 31 | 6 | 319 | 380 | 44 | 51 | 8 | 530 | 618 |
|  |  |  |  |  |  |  |  |  |  |  |  |  |  |  |  |  |  |
| **On concessioned land** |  |  |  |  |  |  |  |  |  |  |  |  |  |  |  |  |  |
| Timber |  |  |  |  |  |  |  |  |  |  |  |  |  |  |  |  |  |
| On reforestation concessions (Sustainable) | 2 | 1,592 | 796 | 39 | 21,514 | 10,757 | 5,892 | 14 | 13 | 7 | 164 | 162 | 21 | 21 | 7 | 259 | 258 |
| On ecotourism concessions ^d^ |  |  |  |  |  |  |  |  |  |  |  |  |  |  |  |  |  |
| Sustainable ^e^ | 5 | 28,371 | 5,674 | 7,346 | 217,447 | 43,489 | 59,132 | 8 | 11 | 4 | 93 | 129 | 17 | 23 | 8 | 202 | 279 |
| Unsustainable (high grading over 5 yrs) ^e^ | 5 | 28,371 | 5,674 | 7,346 | 4,481,221 | 896,244 | 1,402,835 | 158 | 227 | 116 | 689 | 989 | 227 | 326 | 167 | 991 | 1,422 |
| Brazil nuts |  |  |  |  |  |  |  |  |  |  |  |  |  |  |  |  |  |
| On Brazil nut concessions in TNR/BSNP | 67 | 59,780 | 892 | 122 | 140,314 | 2,094 | 284 | 2 | 3 | 0.4 | 28 | 34 | 3 | 3 | 1 | 31 | 37 |
| On Brazil nut concessions across Madre de Dios | 27 | 23,632 | 875 | 302 | 148,604 | 5,504 | 2,950 | 6 | 6 | 1 | 76 | 74 | 7 | 7 | 1 | 80 | 79 |
|  |  |  |  |  |  |  |  |  |  |  |  |  |  |  |  |  |  |

^a^ Derive more than 50% of their revenues from a single crop or fruit or from an animal. Reported numbers include all products produced, not just the specialist products.

^b^ <0.5 and >3.5 animals ha^-1^, and assumes production is only possible over 12 consecutive years (see Muchagata and Brown [77]);

^c^ 0.5-3.5 animals ha^-1^, and assumes production is possible over 25 consecutive years;

^d^ Ecotourism concessions have more and higher-value timber on their lands than do the 29 timber specialists in the household survey.

^e^ In the sustainable format, logging is not conducted on unsuitable land within the ecotourism concessions (e.g., palm swamps), which accounts for lower per hectare profits than in the two sustainable reforestation concessions, which were placed entirely on suitable land. In the unsustainable format, logging is conducted on all land and does not follow the legally mandated 40-year cycle (see Methods).
