# Supplementary material for: The Market Triumph of Ecotourism: An Economic Investigation of the Private and Social Benefits of Competing Land Uses in the Peruvian Amazon
Source: PLoS One. 2010 Sep 29;5(9):e13015. doi: 10.1371/journal.pone.0013015 (PMC2947509; doi:10.1371/journal.pone.0013015)
Supplement: Table S2 — Characteristics of the five inventoried forest areas in Tambopata used to calculate mean commercial timber volume. TF, Terra firme forest; FF, Floodplain forest. The list of commercial timber species used was based on primary information provided by the Forestry and Fauna Department (IFFS) of INRENA in Puerto Maldonado and is also detailed by León-Cornejo and Mego-Canta [1]. (0.06 MB DOC) [file pone.0013015.s002.doc]

**The Market Triumph of Ecotourism: An Economic Investigation of The Private and Social Benefits of Competing Land Uses in the Peruvian Amazon: Supporting Information S2**

Christopher A. Kirkby^1,2,3^, Renzo Giudice-Granados^2^, Brett Day^3^, Kerry Turner^3^, Luz Marina Velarde-Andrade^4^ Agusto Dueñas-Dueñas^5^, Juan Carlos Lara-Rivas^6^ and Douglas W. Yu^1,2,*^

^1^ Ecology, Conservation, and Environment Center (ECEC), State Key Laboratory of Genetic Resources and Evolution, Kunming Institute of Zoology, Chinese Academy of Science, Kunming, Yunnan, China

^2^ Center for Ecology, Evolution and Conservation (CEEC), School of Biological Sciences, University of East Anglia, Norwich, Norfolk, UK

^3^ Center for Social and Economic Research on the Global Environment (CSERGE), School of Environmental Sciences, University of East Anglia, Norwich, Norfolk, UK

^4^ Conservación Ambiental y Desarrollo en el Perú (CAMDE-PERU), Puerto Maldonado, Madre de Dios, Peru

^5^ Cooperazione e Sviluppo (CESVI), Puerto Maldonado, Madre de Dios, Peru.

^6^ Universidad Nacional San Antonio Abad del Cusco (UNSAAC), Puerto Maldonado, Madre de Dios, Peru

* Corresponding author: dougwyu@gmail.com

**Acronyms**

BSNP: Bahuaja-Sonene National Park

PS: producer surplus

INRENA: Instituto Nacional de Recursos Naturales

DBH: diameter at breast height

BAU: business as usual DINAMICA scenario

ECO: ecotourism-led conservation DINAMICA scenario

GPS: geographical positioning system

EEZ: ecological and economic zoning

IOS: Interoceánica Sur Highway

PA: protected areas

NPV: net present value

SPDA: Sociedad Peruana de Derecho Ambiental

TNR: Tambopata National Reserve

**Table S2**. Characteristics of the five inventoried forest areas in Tambopata used to calculate mean commercial timber volume. TF, Terra firme forest; FF, Floodplain forest. The list of commercial timber species used was based on primary information provided by the Forestry and Fauna Department (IFFS) of INRENA in Puerto Maldonado and is also detailed by León-Cornejo and Mego-Canta [1].

| **Location** | **Area (ha)** | **Year** | **Forest type** | **Number of commercial trees** | **Density of commercial trees**  **(trees ha^-1^)** | **Commercial timber density**  **(stumpage volume)**  **(bf ha^-1^)** | **Source** |  |
| --- | --- | --- | --- | --- | --- | --- | --- | --- |
| Explorer’s Inn | 7 | 1994 | TF/FF | 43 | 6.1 | 4,647 | Dr. O. Phillips, Univ. of Leeds | |
| Reserva Amazonica | 4 | 1994 | FF | 38 | 9.5 | 5,926 | Dr. O. Phillips, Univ. of Leeds | |
| Refugio Amazonas | 1 | 2007 | TF | 6 | 6.0 | 2,880 | Collected by author (CK) | |
| Reforestation Concession 1* | 35 | 2005 | TF | 306 | 8.7 | 5,387 | [2] | |
| Reforestation Concession 2** | 42 | 2005 | TF | 309 | 7.4 | 4,964 | [3] | |
| **Mean** | **18** |  |  | **140** | **7.5** | **4,761** |  |  |
| **Total** | **89** |  |  | **702** |  | **23,804** |  |  |

* Reforestation Concession Authorization No. 17-TAM/C-FYR-A-012-04.

** Reforestation Concession Authorization No. 17-TAM/C-FYR-A-001-04.

1. León-Cornejo D, Mego-Canta P (2007) El Cluster forestal en Madre de Dios: obstáculos y oportunidades para su crecimiento y competitividad [Masters thesis]. Lima: Universidad del Pacifico. 62 p.

2. ProNaturaleza (2005a) Plan General de Establecimiento y Manejo Forestal de la Concesion de Reforestacion del Sr. Juan Velasquez Cabrera. Lima: Pronaturaleza. 25 p.

3. ProNaturaleza (2005b) Plan General de Establecimiento y Manejo Forestal de la Concesion de Reforestacion del Sr. Ciro Alagón Huamaní. Lima: Pronaturaleza. 25 p.
