# Supplementary material for: The Market Triumph of Ecotourism: An Economic Investigation of the Private and Social Benefits of Competing Land Uses in the Peruvian Amazon
Source: PLoS One. 2010 Sep 29;5(9):e13015. doi: 10.1371/journal.pone.0013015 (PMC2947509; doi:10.1371/journal.pone.0013015)
Supplement: Table S4 — The mean annual cost (per board foot) associated with managing and extracting timber from reforestation concessions over a 20-yr period (2005–2024). The values do not include (i) payment of extraction rights (US$0.0027 bf−1) and sales taxes (19%) to the government; (ii) depreciation of capital goods (chainsaws, etc.); and (iii) amortization of loans. Based on data from management plans for two reforestation concessions prepared by ProNaturaleza [1], [2]. (0.05 MB DOC) [file pone.0013015.s004.doc]

**The Market Triumph of Ecotourism: An Economic Investigation of The Private and Social Benefits of Competing Land Uses in the Peruvian Amazon: Supporting Information S4**

Christopher A. Kirkby^1,2,3^, Renzo Giudice-Granados^2^, Brett Day^3^, Kerry Turner^3^, Luz Marina Velarde-Andrade^4^ Agusto Dueñas-Dueñas^5^, Juan Carlos Lara-Rivas^6^ and Douglas W. Yu^1,2,*^

^1^ Ecology, Conservation, and Environment Center (ECEC), State Key Laboratory of Genetic Resources and Evolution, Kunming Institute of Zoology, Chinese Academy of Science, Kunming, Yunnan, China

^2^ Center for Ecology, Evolution and Conservation (CEEC), School of Biological Sciences, University of East Anglia, Norwich, Norfolk, UK

^3^ Center for Social and Economic Research on the Global Environment (CSERGE), School of Environmental Sciences, University of East Anglia, Norwich, Norfolk, UK

^4^ Conservación Ambiental y Desarrollo en el Perú (CAMDE-PERU), Puerto Maldonado, Madre de Dios, Peru

^5^ Cooperazione e Sviluppo (CESVI), Puerto Maldonado, Madre de Dios, Peru.

^6^ Universidad Nacional San Antonio Abad del Cusco (UNSAAC), Puerto Maldonado, Madre de Dios, Peru

* Corresponding author: dougwyu@gmail.com

**Acronyms**

BSNP: Bahuaja-Sonene National Park

PS: producer surplus

INRENA: Instituto Nacional de Recursos Naturales

DBH: diameter at breast height

BAU: business as usual DINAMICA scenario

ECO: ecotourism-led conservation DINAMICA scenario

GPS: geographical positioning system

EEZ: ecological and economic zoning

IOS: Interoceánica Sur Highway

PA: protected areas

NPV: net present value

SPDA: Sociedad Peruana de Derecho Ambiental

TNR: Tambopata National Reserve

**Table S4.** The mean annual cost (per board foot) associated with managing and extracting timber from reforestation concessions over a 20-yr period (2005-2024). The values do not include (i) payment of extraction rights (US$0.0027 bf^-1^) and sales taxes (19%) to the government; (ii) depreciation of capital goods (chainsaws, etc.); and (iii) amortization of loans. Based on data from management plans for two reforestation concessions prepared by ProNaturaleza [1,2].

| **Cost variables** | **US$_(2005)_ bf^-1^** |
| --- | --- |
| Forest inventory and monitoring | 0.0024 |
| Opening and maintenance of trails and logging tracks | 0.0040 |
| Timber extraction | 0.0971 |
| Maintenance of seedling nursery and reforestation | 0.0050 |
| Prepare or update annual management plan | 0.0027 |
| **Total** | **0.1112** |

1. ProNaturaleza (2005a) Plan General de Establecimiento y Manejo Forestal de la Concesion de Reforestacion del Sr. Juan Velasquez Cabrera. Lima: Pronaturaleza. 25 p.

2. ProNaturaleza (2005b) Plan General de Establecimiento y Manejo Forestal de la Concesion de Reforestacion del Sr. Ciro Alagón Huamaní. Lima: Pronaturaleza. 25 p.
