# Supplementary material for: The Market Triumph of Ecotourism: An Economic Investigation of the Private and Social Benefits of Competing Land Uses in the Peruvian Amazon
Source: PLoS One. 2010 Sep 29;5(9):e13015. doi: 10.1371/journal.pone.0013015 (PMC2947509; doi:10.1371/journal.pone.0013015)
Supplement: Table S5 — Area and productivity characteristics of the sampled Brazil nut concessions in Tambopata, associated with the Tambopata National Reserve (TNR) and Bahuaja-Sonene National Park (BSNP), and the Economic sample for which financial information on revenues, costs and pre-tax profits was available. (0.04 MB DOC) [file pone.0013015.s005.doc]

**The Market Triumph of Ecotourism: An Economic Investigation of The Private and Social Benefits of Competing Land Uses in the Peruvian Amazon: Supporting Information S5**

Christopher A. Kirkby^1,2,3^, Renzo Giudice-Granados^2^, Brett Day^3^, Kerry Turner^3^, Luz Marina Velarde-Andrade^4^ Agusto Dueñas-Dueñas^5^, Juan Carlos Lara-Rivas^6^ and Douglas W. Yu^1,2,*^

^1^ Ecology, Conservation, and Environment Center (ECEC), State Key Laboratory of Genetic Resources and Evolution, Kunming Institute of Zoology, Chinese Academy of Science, Kunming, Yunnan, China

^2^ Center for Ecology, Evolution and Conservation (CEEC), School of Biological Sciences, University of East Anglia, Norwich, Norfolk, UK

^3^ Center for Social and Economic Research on the Global Environment (CSERGE), School of Environmental Sciences, University of East Anglia, Norwich, Norfolk, UK

^4^ Conservación Ambiental y Desarrollo en el Perú (CAMDE-PERU), Puerto Maldonado, Madre de Dios, Peru

^5^ Cooperazione e Sviluppo (CESVI), Puerto Maldonado, Madre de Dios, Peru.

^6^ Universidad Nacional San Antonio Abad del Cusco (UNSAAC), Puerto Maldonado, Madre de Dios, Peru

* Corresponding author: dougwyu@gmail.com

**Acronyms**

BSNP: Bahuaja-Sonene National Park

PS: producer surplus

INRENA: Instituto Nacional de Recursos Naturales

DBH: diameter at breast height

BAU: business as usual DINAMICA scenario

ECO: ecotourism-led conservation DINAMICA scenario

GPS: geographical positioning system

EEZ: ecological and economic zoning

IOS: Interoceánica Sur Highway

PA: protected areas

NPV: net present value

SPDA: Sociedad Peruana de Derecho Ambiental

TNR: Tambopata National Reserve

**Table S5.** Area and productivity characteristics of the sampled Brazil nut concessions in Tambopata, associated with the Tambopata National Reserve (TNR) and Bahuaja-Sonene National Park (BSNP), and the Economic sample for which financial information on revenues, costs and pre-tax profits was available.

| **Sample** | **N** | **Area (ha)** | | | | **Productivity (kg)** | | | | **Area productivity (kg ha^-1^)** | | | |
| --- | --- | --- | --- | --- | --- | --- | --- | --- | --- | --- | --- | --- | --- |
|  |  | **Total** | **Mean** | **±** | **95%CI** | **Total** | **Mean** | **±** | **95%CI** | **Total** | **Mean** | **±** | **95%CI** |
| Tambopata concessions | 67 | 59,780 | 892 | **±** | 122 | 411,348 | 6,140 | **±** | 834 | 6.9 | 8.2 | **±** | 1.3 |
| Economic concessions | 27 | 23,632 | 875 | **±** | 302 | 377,630 | 13,986 | **±** | 5,184 | 16.0 | 17.1 | **±** | 3.0 |
